# A General Approach for the Synthesis of Arylxenonium(II) Tetrafluoroborates

**DOI:** 10.1021/acs.inorgchem.5c03310

**Published:** 2025-10-23

**Authors:** Pablo Cortés Soláns, Moritz L. Bubenik, Michael H. Lee, Paulin S. Riemann, Alberto Pérez-Bitrián

**Affiliations:** Institut für Chemie, 9373 Humboldt-Universität zu Berlin, Brook-Taylor-Straße 2, Berlin 12489, Germany

## Abstract

Arylxenonium­(II) salts are the most numerous group of organoxenon compounds, i.e., those containing Xe–C bonds, and must be prepared by different routes that lack universality. Here, we present a general synthetic approach to arylxenonium­(II) tetrafluoroborates [RXe]­[BF_4_] containing different degrees of fluorination at the aryl group R via processes that do not require the use of reactive gases or special equipment. The new synthetic access relies on the generation of aryldifluoroboranes RBF_2_ in dichloromethane solution by means of the reaction of K­[RBF_3_] and BF_3_·OEt_2_, which are then transformed in a subsequent step to [RXe]­[BF_4_] salts. This route is not only more accessible than previously existing methods but also yields arylxenonium­(II) tetrafluoroborates in higher yield and purity. Nevertheless, for highly acidic aryldifluoroborane species, namely those with R = C_6_F_5_, C_6_HF_4_, coordination of diethyl ether to the boron center prevents the transfer of the R group to the xenon atom. In these cases, a one-pot procedure starting from BR_3_, XeF_2_ and BF_3_·OEt_2_ enables their easy synthesis, including that of the novel compound [(2,3,5,6-C_6_HF_4_)­Xe]­[BF_4_]. By means of the analysis of ^11^B NMR spectra of the aryldifluoroborane mixtures and theoretical calculations, the design of new organoxenonium­(II) tetrafluoroborates through one route or the other can be tackled.

## Graphical abstract

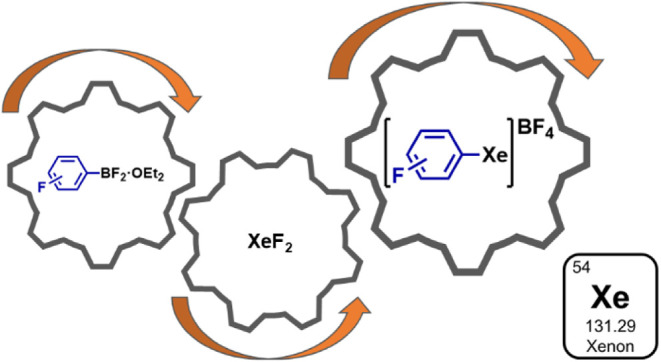

## 1 Introduction

Organoxenon chemistry began its journey in 1989, when the groups of Frohn and Naumann independently reported the first unambiguous example of a compound containing a Xe–C bond.^1,2^ Their synthetic approach to the [C_6_F_5_Xe]^+^ ion was based on the reaction of XeF_2_ and B­(C_6_F_5_)_3_, leading to salts containing different [(C_6_F_5_)_*n*_BF_4–*n*_]^−^ counterions (*n* = 1, 2, 3), depending on the reaction conditions.^1–3^ This milestone in the field of noble gas chemistry set the basis for the development of organoxenon chemistry, which flourished in the next two decades, making available a diversity of compounds containing different types of organic groups attached to the Xe atom.^4–7^ Salts of the organoxenonium­(II) [RXe]^+^ ions constitute the most structurally diverse family, with R being aryl, alkenyl, or alkynyl groups, and among which arylxenonium­(II) salts are the most abundant.^4,7,8^ Importantly, the organic moiety R must be electron-withdrawing to prevent oxidation by Xe^II^. For aryl fragments, this can be readily accomplished by the incorporation of substituents such as F, Cl, CF_3_, or NO_2_.^4,7^

Different synthetic procedures for arylxenonium­(II) salts were reported in the literature in the decades of the 90s and 2000s.^4,7,8^ Direct substitution of hydrogen in arenes was realized thanks to the strong electrophilic Xe­(OCOCF_3_)­(OSO_2_CF_3_), which is able to attack even deactivated benzenes.^9,10^ Nevertheless, this so-called *xenodeprotonation* process is characterized by low yields and a lack of regiospecificity. The one-pot synthesis of [RXe]­[BF_4_] salts by mixing BR_3_, XeF_2_, and BF_3_·OMe_2_, as an improvement of the original synthetic route,^1,2^ entails a straightforward pathway due to its simplicity, although it normally leads only to moderate isolated yields and requires the availability of the corresponding triarylborane BR_3_.^11,12^ The advantage here is the direct generation of the [BF_4_]^−^ anion, which, in comparison to the arylfluoroborates, shows a decreased nucleophilicity and therefore increases the thermal stability of the corresponding salt. A more general route, also applicable to R = alkenyl and alkynyl, involves the in situ generation of aryldifluoroboranes RBF_2_, which can then be reacted with XeF_2_ affording the corresponding [RXe]­[BF_4_] salts.^13^ This procedure, in which the BF_2_ moiety in RBF_2_ is substituted by Xe^+^ derived from XeF_2_ and is normally referred to as *xenodeborylation*,^8^ has been used not only to prepare [RXe]­[BF_4_] species but also the only organoxenonium­(IV) derivative [(C_6_F_5_)­XeF_2_]­[BF_4_].^14,15^ From the experimental point of view, although the procedure is well-established, the synthesis of RBF_2_ requires special material, including vessels made of fluorinated polymers, as well as the inconvenience of bubbling gaseous BF_3_ into the reaction solution for prolonged periods of time.^16–19^

Due to the compatibility of an ether adduct of BF_3_ with the strong-oxidizing reaction mixture, we envisioned a combination of the last two described synthetic routes in a way that would circumvent the use of gaseous BF_3_ and the specialized fluoropolymer-based material traditionally required for the generation of aryldifluoroboranes. Herein, we present a general synthetic approach to arylxenonium­(II) tetrafluoroborate salts through accessible and straightforward routes, which is dependent on the degree of fluorination of the aryl group. Additionally, by means of analysis of the ^11^B chemical shifts of RBF_2_ species, together with computational calculations, a predictive framework for the tailored synthesis of organoxenonium­(II) salts is developed.

## 2 Results and Discussion

### 2.1 Generation of Aryldifluoroboranes in Solution

Our envisioned synthesis relies on the in situ generation of aryldifluoroboranes by means of BF_3_·OEt_2_, which can then be reacted with XeF_2_ to afford the corresponding arylxenonium(II) tetrafluoroborates [RXe]­[BF_4_] (R = fluorinated aryl group). This way, gaseous BF_3_ and all specialized equipment needed for its handling would be avoided. Aiming at showcasing a broad scope, we selected a set of aryl moieties containing one to five fluorine atoms and different substitution patterns (see Figure 1). For the sake of completion, the non-fluorinated phenyl group was also included. The reaction of potassium aryltrifluoroborate salts K­[RBF_3_] (R = aryl group) with an under-stoichiometric amount of BF_3_·OEt_2_ in dichloromethane leads to the formation of the corresponding aryldifluoroborane species in solution. After filtration to separate the solid residue containing K­[BF_4_] and unreacted K­[RBF_3_], a clean solution of the desired aryldifluoroborane is obtained, which is suitable for further synthetic purposes. The use of an excess of K­[RBF_3_] ensures the consumption of BF_3_·OEt_2_, which otherwise, in combination with XeF_2_, is able to act as a one-electron oxidizer, resulting in the fluorination and dearomatization of highly fluorinated aromatic rings.^20–25^ Additionally, due to its extremely low solubility in CH_2_Cl_2_, separation of the precursor excess is facilitated.

**Figure 1:**
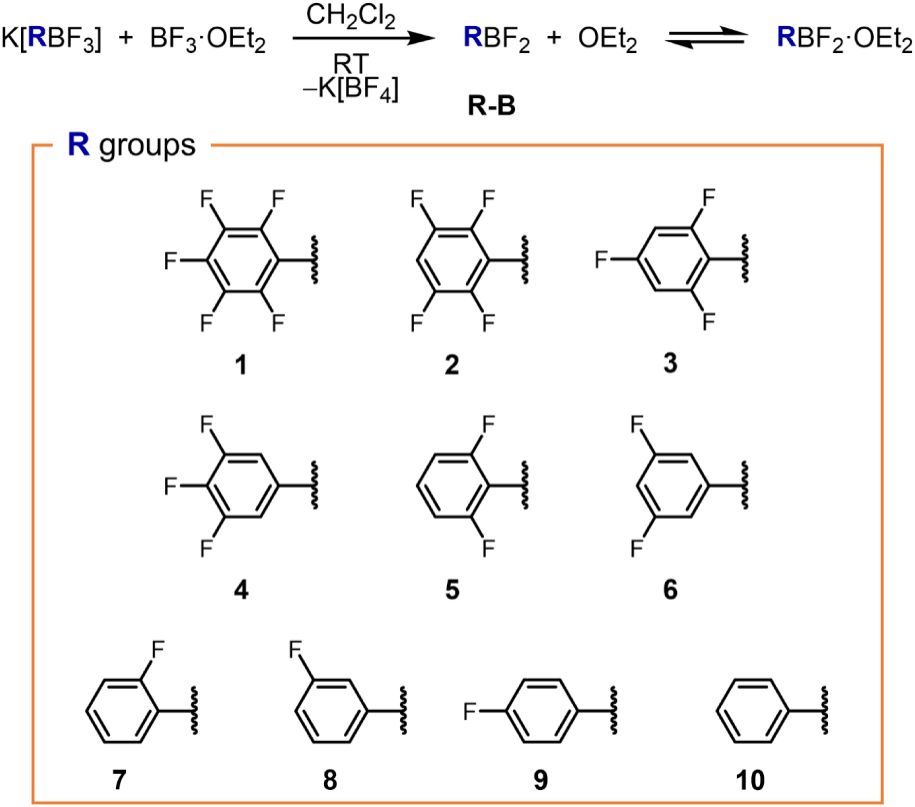
Generation of aryldifluoroboranes RBF_2_ in CH_2_Cl_2_ solution, scope of aryl groups R, and numbering code.

Total conversion of BF_3_·OEt_2_ was confirmed by ^11^B and ^19^F NMR spectroscopy, all spectra showing the expected pattern for the desired RBF_2_ species (see Supporting Information for further details). Interestingly, the ^11^B NMR spectra of the solutions exhibit signals that do not correspond to the free RBF_2_ species, when comparisons can be made (Table 1). All fluorinated species show resonances at lower chemical shifts than the reported values obtained when generating the RBF_2_ compounds by the reaction of K­[RBF_3_] salts with gaseous BF_3_. For the tetrafluoro- and pentafluorophenyl compounds, the only resonance in the ^11^B NMR spectrum appears at *δ*_B_ ≈ 5 ppm, in the region of four-coordinate boron centers. As a comparison, BF_3_ appears at *δ*_B_ = 10.8 ppm in CFCl_3_,^26^ whereas the signal of BF_3_·OEt_2_ (the standard reference in ^11^B NMR) appears at *δ*_B_ = 0.00 ppm, and the ^11^B resonance of B­(4-C_6_F_4_H)_3_ shifts from *δ*_B_ = 59.0 ppm to δ_B_ = 39.3 ppm upon coordination of Et_2_O.^27^ Focusing on aryldifluoroboranes, C_6_F_5_BF_2_·OEt_2_ was assumed to be formed by a similar reaction to that described here as the intermediate in the formation of the 4-phenylpyridine *N*-oxide–BF_2_C_6_F_5_ complex, yet no spectroscopic data were provided.^28^ All in all, Et_2_O coordination is presumably an effect of the higher acidity at the boron center as a consequence of the more electron-withdrawing organic group (*vide infra*).

It is evident from Table 1 that the signal gets more shielded with respect to that of the free Lewis acid RBF_2_ as the fluorination degree of the aryl ring increases from mono- to trifluorinated-substituted aryls. The lower fluorine content of the aryl ring leads to a diminished electron-withdrawing character and, consequently, to a lower Lewis acidity of the borane. This effect accounts for the steady deshielding of the ^11^B resonance, approaching that of the free aryldifluoroborane as the fluorine content decreases. This signifies the existence of an equilibrium between the free borane and the Et_2_O adduct, which is too fast on the NMR time scale to show two separate signals. This quick equilibrium is also evidenced by the broad signals arising from the -B*F*_2_ nuclei in the ^19^F NMR spectra, indicative of a dynamic situation. These signals also appear, as expected, at different *δ*_F_ than those of the free RBF_2_ compounds (see Supporting Information). The non-fluorinated species C_6_H_5_BF_2_ (**10-B**) exhibits a resonance at *δ*_B_ = 24.6 ppm,^29^ fitting almost exactly the one obtained through the BF_3_·OEt_2_ approach (*δ*_B_ = 22.95 ppm). Interestingly, Lewis acid C_6_H_5_BF_2_ was claimed to be prepared through our procedure,^30^ yet the reported *δ*_B_ = 21.88 ppm virtually fits better to our value than to that of the free Lewis acid, indicating that, although weakly, the Et_2_O might still interact with the boron center.

**Table 1:** ^11^B NMR Chemical Shifts of Aryldifluoroboranes RBF_2_ Obtained by Using the BF_3_·OEt_2_ Approach (This Work) and the BF_3_ Route

	*δ*_B_ [ppm]	
R group	BF_3_·OEt_2_ approach^a^	BF_3_ approach
**C**_**6**_ **F**_**5**_	5.15	22.84^b^
**2,3,5,6-C**_**6**_ **HF**_**4**_	5.18	^c^
**2,4,6-C**_**6**_ **H**_**2**_ **F**_**3**_	8.80	23.26^b^
**3,4,5-C**_**6**_ **H**_**2**_ **F**_**3**_	12.14	23.88^b^
**2,6-C**_**6**_ **H**_**3**_ **F**_**2**_	8.88	23.53^b^
**3,5-C**_**6**_ **H**_**3**_ **F**_**2**_	12.99	24.04^b^
**2-C**_**6**_ **H**_**4**_ **F**	15.21	24.23^b^
**3-C**_**6**_ **H**_**4**_ **F**	15.08	24.52^b^
**4-C**_**6**_ **H**_**4**_ **F**	20.57	24.69^b^
**C**_**6**_ **H**_**5**_ ^d^	22.95	24.6^e^

a All spectra were recorded in CD_2_Cl_2_ solution at room temperature (see Supporting Information for complete data sets).

b Data in CD_2_Cl_2_ at 35 °C, as taken from ref. 16.

c Not reported.

d Compound C_6_H_5_BF_2_ was reported to be prepared by the reaction of K­[(C_6_H_5_)­BF_3_] with gaseous BF_3_ at low temperatures, but no characterization data were provided; see ref. 18.

e Chemical shifts in CDCl_3_ corresponding to compound C_6_H_5_BF_2_ prepared by the reaction of C_6_H_5_BCl_2_ and Na­[BF_4_]; see ref. 29.

### 2.2 Synthesis of Arylxenonium­(II) Tetrafluoroborates

With the solutions of the aryldifluoroborane compounds in hand, the corresponding arylxenonium­(II) tetrafluoroborates should be accessible via a straightforward reaction with XeF_2_, in a similar process to that described by the group of Frohn.^13^ Due to the high reactivity and instability of the formed products, all reactions were performed in thoroughly dried glassware at low temperatures. The dropwise addition of the previously cooled solution of the aryldifluoro­borane to a saturated solution of XeF_2_ in CH_2_Cl_2_ at −40 °C afforded the corresponding [RXe]­[BF_4_] compounds **3-Xe**–**9-Xe** as white powders that precipitated immediately from the reaction mixture (Figure 2). Surprisingly, whereas mono-, di-, and trifluorophenyl moieties could be easily transferred to the xenon center, for tetrafluoro- and pentafluorophenylxenonium­(II) salts, no precipitate was observed and, in fact, XeF_2_ was recovered after evaporation of volatiles. Reaction mixtures additionally contained varied unidentified side products.

**Figure 2:**
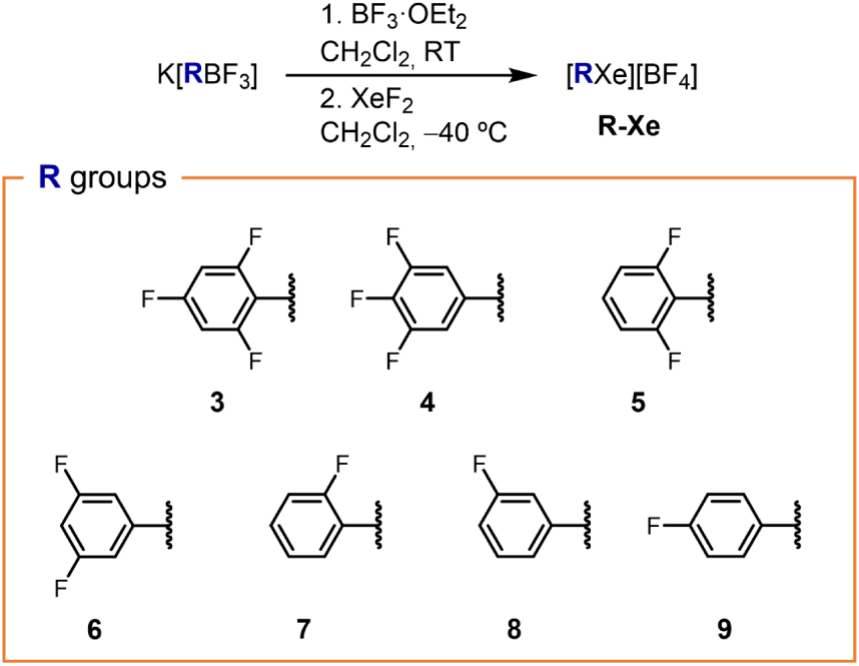
Synthesis of arylxenonium­(II) tetrafluoroborates [RXe]­[BF_4_] by means of in situ generated aryldifluoroboranes RBF_2_ by using the BF_3_·OEt_2_ approach (see Figure 1), scope of aryl groups R, and numbering code.

Owing to the high insolubility of these compounds in dichloromethane, the purity of the samples was confirmed by ^1^H and ^19^F NMR spectroscopy in CD_3_CN solution (see Supporting Information). The ^19^F NMR signals of the *ortho*-F atoms of compounds **3-Xe**, **5-Xe**, and **7-Xe** show satellites due to the coupling to the NMR-active ^129^Xe isotope, similarly to the *ortho*-H in compounds **4-Xe**, **6-Xe**, and **7-Xe**. In compounds **8-Xe** and **9-Xe**, the low coupling constants result in these satellites not being visible. Within the first set of compounds,^3^ *J*_F–Xe_ are rather similar, with a value of approximately 50 Hz, and the satellites can be undoubtedly seen flanking the main signal. For the second set, the coupling constants ^3^ *J*_H–Xe_ are of approximately 20 Hz, therefore not appearing completely resolved as satellites but as shoulders of the main signal instead.

All compounds **3-Xe**–**9-Xe** were obtained as white solids and can be stored in perfluoroalkoxy alkane (PFA) containers in the glovebox at −35 °C for months (**3-Xe**, **5-Xe**, **7-Xe**) or weeks (**4-Xe**, **6-Xe**) without apparent signs of decomposition. The more unstable monofluorinated species **8-Xe** and **9-Xe** could only be handled at temperatures below −40 °C, and therefore similar storage conditions as in the other cases were not possible. Unfortunately, [C_6_H_5_Xe]­[BF_4_] could not be prepared by the same procedure, which is not unexpected. In fact, calculated charges showed that the lack of electron-withdrawing substituents in the aryl group hinders the generation of a negative charge in the *ipso*-carbon able to stabilize the Xe–C bond.^31^

This approach offers significant improvements to those reported previously in the literature. First, the setups required to perform the reactions described here are accessible by a trained synthetic inorganic chemist. Second, it does not require the use of corrosive gases (such as BF_3_) and, in fact, all reactions can be performed in glassware, as long as it is properly dried to avoid the presence of moisture. This also implies a significant reduction in the required amount of fluoropolymer, yet containers made of materials such as perfluoroalkoxy alkanes (PFA) are needed to store the compounds for prolonged periods of time once synthesized. Additionally, focusing on the monofluorinated species **7-Xe**–**9-Xe**, this method provides a significant improvement when compared to the literature-reported syntheses, which rely on the reaction of XeF_2_ with triaryl boranes BR_3_. In the case of the *ortho-*substituted **7-Xe**, a very low yield of 7% was reported,^11^ which is now improved to 95% through our approach. This same method rendered the *para-*compound **9-Xe** in 53% yield,^11^ yet it is now increased to 77%. The *meta-*substituted [(3-C_6_H_4_F)­Xe]^+^ cation has now been isolated in pure form as the tetrafluoroborate salt **8-Xe**. This is also a significant improvement, since this cation had only been reported to be obtained with a mixture of borate anions [(3-C_6_FH_4_)­BF_3_]^−^ and [BF_4_]^−^, which could then be transformed into the tetrafluoroborate salt by bubbling BF_3_ into the reaction mixture.^32^ Similar results were reported for the *para-*substituted species,^32^ whose characterization was initially problematic.^33^ Therefore, our method enables the synthesis of these three compounds as pure substances containing only the [BF_4_]^−^ anion in high yield and purity.

Compounds **3-Xe** and **5-Xe** were only reported to be prepared by means of the reaction of the corresponding BR_3_, XeF_2_, and BF_3_·OMe_2_,^11,12^ whereas for **4-Xe** and **6-Xe**, only the *xenodeborylation* path starting from RBF_2_ has been described.^13^ For these species, our yields still surpass previously reported values in most cases, though they are not as high as those attained for the monofluorinated species. The lower isolated yields for the higher fluorinated compounds are attributed to their higher solubility in CH_2_Cl_2_ with respect to the monofluorinated species, which implies the loss of some material during the workup of the reactions, i.e., when washing the salts with cold CH_2_Cl_2_ (see Supporting Information).

Unfortunately, the synthesis of the highly fluorinated arylxenonium­(II) tetrafluoroborates via this route turned out to be unfeasible, presumably because of the strong coordination of the Et_2_O molecule to the RBF_2_ species (*vide infra*). Other approaches to generate RBF_2_ species without the need for gaseous BF_3_ were also considered,^34–36^ but they were dismissed due to the incompatibility of the solvent with the subsequent reaction with XeF_2_ or with the formed arylxenonium­(II) tetrafluoroborate.

Aiming at developing a full set of arylxenonium­(II) tetrafluoroborates covering also high fluorination degrees at the phenyl ring, as is the case of **1-Xe** and **2-Xe**, we resorted to the original idea from Naumann, namely the one-pot reaction of the triaryl borane BR_3_, XeF_2_, and BF_3_·OEt_2_ (see Figure 3a). Although the very first synthesis of the [C_6_F_5_Xe]^+^ ion relied on the reaction of B­(C_6_F_5_)_3_ with XeF_2_, [C_6_F_5_Xe]­[BF_4_] was never reported to be prepared following this approach, i.e., with the addition of BF_3_·OEt_2_. This is surprising, especially since all of the starting materials are commercially available. Gratifyingly, the reaction worked smoothly and actually allowed us to obtain compound **1-Xe** in high purity and a yield close to 50%. Our approach leads to **1-Xe** as a pure substance, containing exclusively [BF_4_]^−^ as the anion, as was the case for compounds **3-Xe**, **5-Xe**, **7-Xe,** and **9-Xe** when prepared in a similar manner.^11,12^ A related route was reported to be performed in anhydrous HF, allowing the transfer of all pentafluorophenyl groups of B­(C_6_F_5_)_3_, yet the [C_6_F_5_Xe]^+^ cation is obtained in this case as a mixed salt containing the anions [BF_4_]^−^ and [HF_2_]^−^.^37^

**Figure 3:**
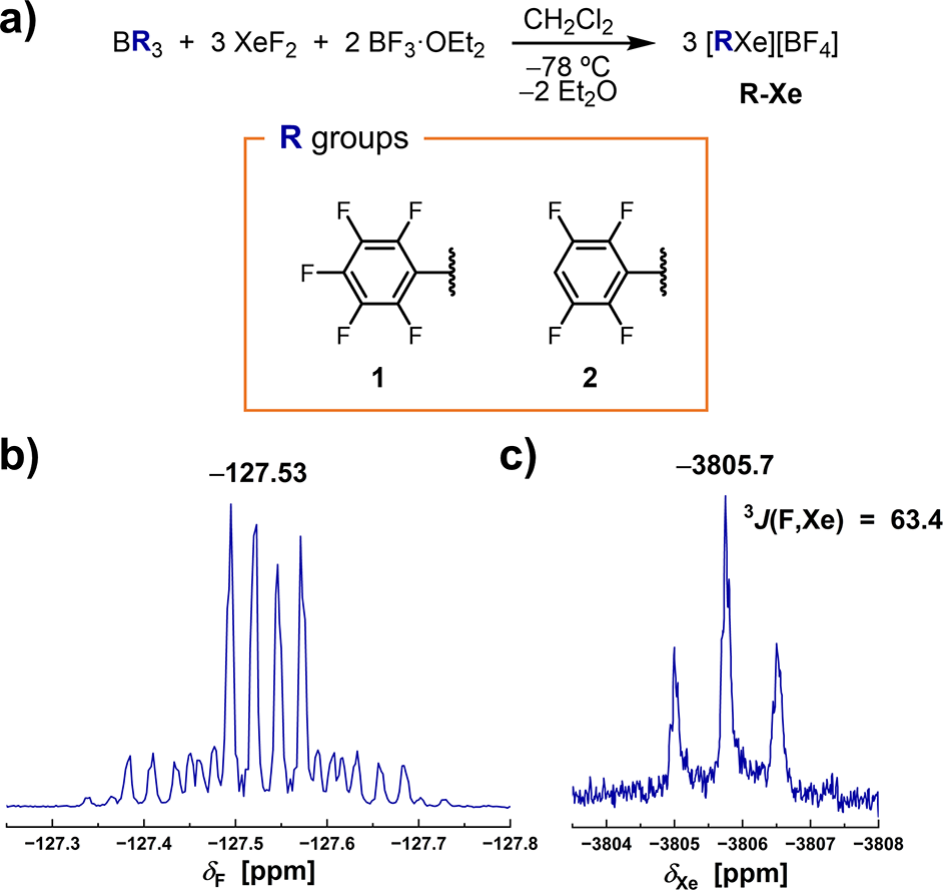
(a) Synthesis of arylxenonium­(II) tetrafluoroborates [RXe]­[BF_4_] by means of the one-pot synthesis using triaryl boranes BR_3_, scope of new aryl groups R and numbering code. (b) Section of the ^19^F NMR spectrum (282.40 MHz, CD_3_CN, 298 K) of **2-Xe** depicting the *ortho*-F atoms (with *δ*_F_ in ppm indicated) of the tetrafluorophenyl moiety, showing the corresponding ^129^Xe satellites. (c) ^129^Xe NMR spectrum (83.47 MHz, CD_3_CN, 298 K) of **2-Xe** depicting a triplet due to the coupling to the two *ortho*-F atoms of the tetrafluorophenyl moiety (*δ*_Xe_ in ppm, *J* in Hz).

Following a similar synthetic route, the tetrafluorophenylxenonium­(II) salt [(2,3,5,6-C_6_HF_4_)­Xe]­[BF_4_] (**2-Xe**) was prepared. This unprecedented compound was obtained as a white powder that is stable at room temperature for similar periods of time as the pentafluorophenyl analogue **1-Xe** and can therefore be stored in a similar manner, namely, in a PFA tube for months. The nature of **2-Xe** was proved by multinuclear NMR spectroscopy. First, in the ^19^F NMR spectrum, in addition to the signal corresponding to the [BF_4_]^−^ anion, two signals corresponding to the two types of chemically inequivalent fluorine nuclei appear at −127.53 ppm and −133.53 ppm. The former can be assigned to the *ortho*-F, which displays ^129^Xe satellites flanking the main signal demonstrating that the aryl group is coordinated to the xenon center (Figure 3b), and the latter to the *meta*-F. The coupling constant ^3^ *J*_F–Xe_ = 63.4 Hz is only slightly smaller than that reported for [C_6_F_5_Xe]­[BF_4_] under similar measuring conditions (66.6 Hz),^38^ but virtually the same as that of the other known tetrafluorophenylxenonium­(II) tetrafluoroborate [(2,3,4,5-C_6_HF_4_)­Xe]­[BF_4_].^13^ In the ^129^Xe NMR, a triplet at –3805.7 ppm indicates the coupling to the two fluorine atoms in the *ortho* positions of the phenyl group (Figure 3c). As expected, the δ_Xe_ is rather similar to that of [C_6_F_5_Xe]­[BF_4_] (−3802.8 ppm).^38^ Crystallization attempts to determine the molecular structure in the solid state by X-ray diffraction have failed thus far.

### 2.3 The Role of Et_2_O and the Lewis Acidity of Aryldifluoroboranes

Both synthetic routes described in this work lie within the so-called *xenodeborylation* process, which entails the acid-assisted transfer of an R group from RBX_2_ species (R = fluorinated aryl group; X = F, R) to the xenon center of XeF_2_.^8^ The course of this process has been discussed since the reaction was first described. In the first step, the Xe–F bond has to be polarized for the reaction to start—as otherwise, the lack of a permanent dipole moment in XeF_2_ complicates the reaction kinetically. This is normally achieved by the interaction of one of the basic fluorine atoms with a boron center, therefore leading to the asymmetry of the 3c–4e bond of XeF_2_. Boranes are suitable in this regard since they do not lead to the complete abstraction of the fluoride; yet instead, tetracoordination at the boron center is achieved. This way, the electrophilicity of Xe­(II) is enhanced, while at the same time, the nucleophilicity of the *ipso*-carbon of the organic group R at the boron center increases, resulting in the substitution of one F by the R group. The resulting borane is then able to abstract a fluoride ion from the neutral RXeF, therefore leading to the formation of the [RXe]­[BF_2_X_2_] salt.

In the course of our investigations, and as described above, we have found that the nature of the organic group was decisive for the suitability of our novel synthetic approach to arylxenonium­(II) tetrafluoroborates. To better understand the feasibility of the route depicted in Figure 2 and give some predictive rules for its use, we turned our attention to the key species involved in the process: aryldifluoroboranes RBF_2_. For this synthesis to succeed, one F in XeF_2_ has to be able to interact with the boron center of the corresponding borane, as just described. This means that, by using BF_3_·OEt_2_, the Et_2_O molecule has to be as weakly coordinated as possible to the boron center, in a way that interaction with the F atom of XeF_2_ is energetically more favorable. Having a look at the NMR data for the species resulting from the reaction of K­[RBF_3_] with BF_3_·OEt_2_, it is clear that the signal in the ^11^B NMR, normally within a very narrow range of approximately 2 ppm for pure RBF_2_, exhibits now a much broader variation of nearly 20 ppm (see Table 1). This observation was already rationalized by the strength of the interaction of the diethyl ether molecule with the borane, which is therefore dependent on its Lewis acidity. In fact, the Lewis acidity of the aryldifluoroborane has been described in the literature as one of the main factors governing the synthesis of organoxenonium­(II) ions from boranes.^39^ According to our NMR data (see Table 1), the highly fluorinated boranes **1-B** and **2-B** are obtained as their Et_2_O adducts, with *δ*_B_ of approximately 5 ppm, indicating tetracoordination at the boron atom. On the contrary, the lower fluorinated species coordinate Et_2_O in a more labile way, with *δ*_B_ increasing as the degree of fluorination decreases, and therefore allow the interaction of a Xe–F moiety of XeF_2_ with the borane.

To shed light on this phenomenon, we decided to investigate the Lewis acidity of these aryldifluoroboranes by a theoretical study. First, we calculated the bond dissociation energies for the Et_2_O adducts, *D*_e_(B–OEt_2_), which are collected in Table 2. For the sake of comparison, we included the BF_3_·OEt_2_ adduct, for which *D*_e_(B–OEt_2_) = 45.2 kJ mol^–1^. Interestingly, both **1-B** and **2-B** exhibit the same bond dissociation energy of 38.4 kJ mol^–1^. Within our set of compounds, it seems that a *D*_e_(B–OEt_2_) below 30 kJ mol^–1^ enables the dissociation of the Et_2_O molecule sufficiently for XeF_2_ to interact. Interestingly, the synthesis of [RXe]­[BF_4_] starting from BR_3_ (R = 2,6-C_6_H_3_F_2_, 2-C_6_H_4_F, and 4-C_6_H_4_F), XeF_2_, and BF_3_·OMe_2_ was suggested to proceed via the formation of an aryldifluoroborane RBF_2_ species as an (undetectable) intermediate.^11^ This would, in principle, seem viable since the Et_2_O present in the reaction mixture should be labile enough to lead to the transfer of the R group. Nevertheless, since we have demonstrated that this synthesis works also for R = C_6_F_5_ and 2,3,5,6-C_6_HF_4_, whose synthesis does not proceed when there is Et_2_O in the reaction medium, a different reaction pathway might be more feasible.

**Table 2:** Bond Dissociation Energies (*D*_e_) and Fluoride Ion Affinities (FIAs) of the Indicated Aryldifluoroboranes RBF_2_ ^a^

R group	*D*_e_(B–OEt_2_) [kJ mol^–1^]	FIA [kJ mol^–1^]^b^
**C**_**6**_ **F**_**5**_	38.4	385.0
**2,3,5,6-C**_**6**_ **HF**_**4**_	38.4	376.1
**2,4,6-C**_**6**_ **H**_**2**_ **F**_**3**_	29.5	353.1
**3,4,5-C**_**6**_ **H**_**2**_ **F**_**3**_	25.8	362.7
**2,6-C**_**6**_ **H**_**3**_ **F**_**2**_	29.4	342.8
**3,5-C**_**6**_ **H**_**3**_ **F**_**2**_	26.1	354.4
**2-C**_**6**_ **H**_**4**_ **F**	26.9	334.9
**3-C**_**6**_ **H**_**4**_ **F**	21.6	337.3
**4-C**_**6**_ **H**_**4**_ **F**	17.4	330.3
**C**_**6**_ **H**_**5**_	17.2	319.9
**F**	45.2	354.5

a DFT gas-phase calculations at the B3LYP-D3BJ/def2-TZVPP level of theory.

b Isodesmic reactions with Me_3_SiF/Me_3_Si^+^ as anchor were used.^40,41^

To provide further insights into the Lewis acidity of these relevant compounds, which add to the body of work of boron-based Lewis acids,^42–44^ their fluoride ion affinities (FIAs) were also calculated (see Table 2). As expected, the degree of fluorination of the aryl group attached to the boron increases the Lewis acidity in general terms. The FIA values of some of these compounds, expressed in the pF^–^ scale,^45^ had been reported some time ago and correlate well with those presented here.^39^ Similarly, the Lewis acidity of some of these species has been assessed previously by means of the Gutmann-Beckett method, providing a similar trend.^46^ Interestingly, as inferred from the data, the Lewis acidity trend varies with the chosen scale for a given degree of fluorination, namely, with the substitution pattern. As an example, for the trifluorophenyl species, the *D*_e_(B–OEt_2_) is higher for (2,4,6-C_6_H_2_F_3_)­BF_2_ yet the FIA is higher for (3,4,5-C_6_H_2_F_3_)­BF_2_. This different trend has already been observed in the particular case of these same aryl groups, but in BR_3_ compounds, when comparing the FIA to the experimentally determined Lewis acidity/basicity scale derived from isothermal titration calorimetry measurements.^47^ Additionally, computational studies on the binding energies of NMe_3_ or PMe_3_ to triarylboranes also demonstrated that the position of the fluorine substituents matters.^48^ Indeed, those energies are significantly affected by the presence of *ortho*-F atoms, where the second substitution exerts a steric factor that opposes the Lewis acidity.

In the case of the aryldifluoroborane species considered here, the bond dissociation energies of the Et_2_O adducts seem to provide a more accurate description for our purpose of their acceptor ability, namely, the lability of this molecule to allow the transfer of the R group to the Xe atom. This proves once again that a three-coordinate borane species is necessary to form an arylxenonium species, either an aryldifluoroborane species, as discussed here, or a triarylborane. At this point, it is worth noting that, in fact, attempts to use tetrahedral or trigonal bipyramidal Lewis acids of moderate strength have been reported to fail.^8^

This experimental (^11^B NMR) and theoretical (*D*_e_(B–OEt_2_)) framework comprises a unique tool for the design of organoxenonium­(II) tetrafluoroborates. For arylxenonium­(II) salts, evaluation of the ^11^B NMR signal of the RBF_2_ species clearly hints toward the viability of the stepwise synthetic route. Although for alkenyls and alkynyls it might be useful, slightly different δ_B_ come into play, and therefore one should better rely on the bond dissociation energy of the Et_2_O molecule from the RBF_2_·OEt_2_ adduct, from which the transfer of the R group can occur, provided that the value is lower than 30 kJ mol^–1^.

## 3 Conclusions

In conclusion, we have developed a synthetic route toward organoxenonium­(II) tetrafluoroborates by substituting gaseous BF_3_ for liquid BF_3_·OEt_2_, therefore allowing the use of glassware instead of more specialized equipment, including more controversial fluorinated polymers. This route is not only more accessible to every laboratory but also affords arylxenonium­(II) tetrafluoroborates in a higher yield and purity. In fact, this has been the first time that a wide array of fluorinated aryl groups of different characteristics has been investigated simultaneously to give a broader view toward a general synthesis.

Unfortunately, this new route does not work for highly fluorinated aryl groups, for which we have expanded the use of the known one-pot synthetic pathway starting from triarylboranes. The limitation of our new approach to aryl groups R containing three or fewer fluorine substituents is due to highly acidic RBF_2_ species being formed as adducts containing a strongly coordinated Et_2_O molecule, which hinders the subsequent reaction with XeF_2_. We were able to explain this key feature of the process both by ^11^B NMR of the in situ formed aryldifluoroborane species, along with the calculated dissociation energy of the Et_2_O from the aryldifluoroborane adduct. This combination of tools provides a framework for the selection of a suitable synthetic route for newly designed organoxenonium­(II) tetrafluoroborates. We hope that this work opens the door to a new era for these unique compounds. Further investigations into these compounds, their properties, and their potential applications are currently ongoing in our laboratory.

## 4 Experimental Section

### 4.1 CAUTION: Strong Oxidizers!

Organoxenon compounds are potentially explosive compounds that might react violently with organic materials due to their strong oxidizing nature. Therefore, they must be handled with extreme care. Contact with metal must be avoided since it can result in immediate, more or less violent decomposition. The synthesis of arylxenonium­(II) tetrafluoroborates described in this work can be carried out in thoroughly dried glassware, but for long-term storage, they must be kept in custom-made PFA containers.

### 4.2 Reaction of K­[RBF_3_] Salts (R = Fluorinated Aryl Group) with BF_3_·OEt_2_

The corresponding K­[RBF_3_] salt (1.5 equiv) was suspended in CH_2_Cl_2_ and cooled to −78 °C. A solution of BF_3_·OEt_2_ (1 equiv) in CH_2_Cl_2_ at −78 °C was added dropwise to the suspension of K­[RBF_3_] at −78 °C. The reaction mixture was allowed to reach room temperature while stirring overnight. After filtration to separate all solids, the filtrate was analyzed by NMR to ensure the complete conversion of BF_3_·OEt_2_. The obtained CH_2_Cl_2_ solution containing the aryldifluoroborane RBF_2_·OEt_2_ (60% yield estimated on further reactions) can be used directly for subsequent steps in the synthesis of [RXe]­[BF_4_] salts **3-Xe**–**9-Xe** (*vide infra*).

### 4.3 Synthesis of [RXe]­[BF_4_] Salts 3-Xe–9-Xe from CH_2_Cl_2_ Solutions of RBF_2_ Species

A freshly prepared CH_2_Cl_2_ solution of the corresponding aryldifluoroborane (1 equiv) at −40 °C was slowly added to a saturated solution of XeF_2_ (1 equiv) in CH_2_Cl_2_ at −40 °C, resulting in the quick formation of the arylxenonium­(II) tetrafluoro­borate, which precipitates from the reaction mixture. The suspension was kept at −86 °C overnight to allow the solid to be deposited. The solvent was removed, and the arylxenonium­(II) tetrafluoroborate [RXe]­[BF_4_] was then washed with CH_2_Cl_2_ at −78 °C and dried under vacuum to obtain the corresponding [RXe]­[BF_4_] as a white powder. Salts **3-Xe**–**9-Xe** were characterized by NMR spectroscopy in CD_3_CN.

### 4.4 One-Pot Synthesis of [RXe]­[BF_4_] Salts 1-Xe and 2-Xe from BR_3_ Compounds

A solution of XeF_2_ (3 equiv) in CH_2_Cl_2_ at −40 °C was added dropwise to a suspension containing B­(C_6_F_5‑*n*_H_*n*_)_3_ (*n* = 0, 1; 1 equiv) and BF_3_·OEt_2_ (2 equiv) in CH_2_Cl_2_ also at −40 °C. The initially colorless solution turned yellow, whereas at the same time a white precipitate appeared. After the addition, the reaction mixture was stirred for 4 h at −78 °C, and then the white solid was allowed to settle at −86 °C. Removal of the solvent and subsequent washing with cold CH_2_Cl_2_ rendered a white residue, which was dried under vacuum to obtain the desired [RXe]­[BF_4_] salt as a white powder. Salts **1-Xe** and **2-Xe** were characterized by NMR in CD_3_CN.

### 4.5 Computational Details

Density Functional Theory (DFT) calculations were performed by using the Gaussian 16 (Revision A.03) software package.^49^ Geometry optimizations were carried out by means of the B3LYP exchange-correlation functional,^50^ in conjunction with the D3BJ dispersion correction scheme^51,52^ and the triple-ζ def2TZVPP Ahlrichs basis set.^53^ The nature of stationary points was confirmed by a vibrational analysis calculation to be energy minima (no imaginary frequencies).

## Supplementary Material



## Notes

General procedures and materials, detailed synthetic procedures for the generation of aryldifluoroboranes in solution and for the synthesis of arylxenonium­(II) tetrafluoroborates, characterization data for all compounds, NMR spectra for all compounds, computational details and Cartesian coordinates of the DFT-optimized structures (PDF)
