# Supplementary material for: A General Approach for the Synthesis of Arylxenonium(II) Tetrafluoroborates
Source: Inorg Chem. 2025 Oct 23;64(47):23184–91. doi: 10.1021/acs.inorgchem.5c03310 (PMC12673524; doi:10.1021/acs.inorgchem.5c03310)
Supplement: Supplementary file 1 [file ic5c03310_si_001.pdf]

Supporting Information

for

A General Approach for the Synthesis of Arylxenonium(II) Tetrafluoroborates

Pablo Cortés Soláns, Moritz L. Bubenik, Michael H. Lee, Paulin S. Riemann, Alberto Pérez-Bitrián*

Institut für Chemie, Humboldt-Universität zu Berlin, Brook-Taylor-Straße 2, 12489 Berlin, Germany.

Email: alberto.perez-bitrian@hu-berlin.de

Table of contents

1. General procedures and materials S2
2. Generation of aryldifluoroboranes in solution S4
3. Synthesis of arylxenonium(II) tetrafluoroborates S8
4. NMR spectra S12
5. Quantum-chemical calculations S31
6. References S43

1. General procedures and materials

All experiments were performed under rigorous exclusion of moisture and oxygen using standard Schlenk techniques. Solids were handled in a MBRAUN UNIlab plus glovebox under an argon atmosphere (O_2 < 0.5 ppm, H_2O < 0.5 ppm). Solvents were dried using a MBraun SPS-800 solvent system (CH_2Cl_2, MeCN, Et_2O, *n*-pentane), or over 3 Å molecular sieves (CD_2Cl_2, CD_3CN) and were deoxygenated before use. K[RBF_3] salts (R = C_6F_5, 2,3,5,6-C_6HF_4, 2,4,6-$C_6H_2F_3$, 3,4,5-$C_6H_2F_3$, 2,6-$C_6H_3F_2$, 3,5-$C_6H_3F_2$, 2-C_6H_4F, 3-C_6H_4F, 4-C_6H_4F) were prepared according to literature procedures,[1,2] as well as triarylborane B(2,3,5,6-C_6HF_4)$_3$.[3] XeF_2 was prepared from a gaseous mixture of xenon and fluorine in a Pyrex flask, exposed to UV light,[4] and was stored inside a glovebox until used. All other reagents were purchased from standard commercial suppliers and used as received. NMR spectra were recorded on a Bruker Avance III 500, a Bruker Avance II 300, a Bruker Avance III 300, or a Bruker Avance NEO 300 MHz spectrometer using the solvent as the internal lock. All reported chemical shifts (*δ* in ppm) are referenced to the Ξ values given in the IUPAC recommendations of 2008 using the ^{2}H signal of the deuterated solvent as internal reference.[5] Multiplicity is indicated as follows: s = singlet, br s = broad singlet, t = triplet, dd = doublet of doublets, tt = triplet of triplets, m = multiplet, sh = shouldered. Elemental analysis could not be performed for any of the samples due to their high moisture- and temperature-sensitive nature, as well as their strong oxidizing properties.

CAUTION strong oxidizers! Organoxenon compounds are potentially explosive compounds which might react violently with organic materials due to their strong oxidizing nature. Therefore, they must be handled with extreme care. Contact with metal must be avoided, since it can result in immediate more or less violent decomposition. The

synthesis of arylxenonium(II) tetrafluoroborates described in this work can be carried out in thoroughly dried glassware, but for long-term storage, they must be kept in custom-made PFA containers.

2. Generation of aryldifluoroboranes in solution

2.1. Reaction of $K[(C_6F_5)BF_3]$ with $BF_3·OEt_2$ to obtain **1-B**

A solution of $BF_3·OEt_2$ (0.30 mL, 2.43 mmol) in CH_2Cl_2 (6 mL) was cooled to −78 °C and added slowly to a suspension of $K[(C_6F_5)BF_3]$ (1.00 g, 3.65 mmol) in CH_2Cl_2 (2 mL) at −78 °C. The resulting suspension was allowed to reach room temperature while stirring overnight. After filtration to separate all solid materials, the obtained CH_2Cl_2 solution containing **1-B** was analyzed by ^{11}B and ^{19}F NMR spectroscopy:

^{11}B NMR (160.46 MHz, CD_2Cl_2, 298 K; Figure S1): δ_B/ppm = 5.15 (br s).

^{19}F NMR (470.59 MHz, CD_2Cl_2, 298 K; Figure S2): δ_F/ppm = −135.25 (m, 2F, *o*-F), −138.95 (br s, 2F, BF_2), −155.50 (t, 1F, $^3J(^{19}F_p, ^{19}F_m)$ = 19.9 Hz, *p*-F), −163.98 (m, 2F, *m*-F).

2.2. Reaction of $K[(2,3,5,6\text{-}C_6HF_4)BF_3]$ with $BF_3·OEt_2$ to obtain **2-B**

By following the procedure described for the generation of **1-B**, a CH_2Cl_2 solution of **2-B** was prepared starting from $K[(2,3,5,6\text{-}C_6HF_4)BF_3]$ (700 mg, 2.73 mmol) in CH_2Cl_2 (5 mL) and $BF_3·OEt_2$ (0.23 mL, 1.82 mmol) in CH_2Cl_2 (2 mL). The obtained CH_2Cl_2 solution containing **2-B** was analyzed by ^{11}B and ^{19}F NMR spectroscopy:

^{11}B NMR (160.46 MHz, CD_2Cl_2, 298 K; Figure S3): δ_B/ppm = 5.18 (br s).

^{19}F NMR (470.59 MHz, CD_2Cl_2, 298 K; Figure S4): δ_F/ppm = −135.74 (br s, 2F, *o*-F), −138.90 (br s, 2F, BF_2), −140.63 (m, 2F, *m*-F).

2.3. Reaction of $K[(2,4,6\text{-}C_6H_2F_3)BF_3]$ with $BF_3·OEt_2$ to obtain **3-B**

By following the procedure described for the generation of **1-B**, a CH_2Cl_2 solution of **3-B** was prepared starting from $K[(2,4,6\text{-}C_6H_2F_3)BF_3]$ (920 mg, 3.87 mmol) in CH_2Cl_2 (4 mL) and $BF_3·OEt_2$ (0.32 mL, 2.58 mmol) in CH_2Cl_2 (1.5 mL). The obtained CH_2Cl_2 solution containing **3-B** was analyzed by ^{11}B and ^{19}F NMR spectroscopy:

^{11}B NMR (160.46 MHz, CD_2Cl_2, 298 K; Figure S5): δ_B/ppm = 8.80 (br s).

^{19}F NMR (470.59 MHz, CD_2Cl_2, 298 K; Figure S6): δ_F/ppm = −101.08 (br s, 2F, *o*-F), −107.46 (m, 1F, *p*-F), −128.41 (br s, 2F, BF_2).

2.4. Reaction of K[(3,4,5-$C_6H_2F_3$)BF_3] with BF_3·OEt_2 to obtain **4-B**

By following the procedure described for the generation of **1-B**, a CH_2Cl_2 solution of **4-B** was prepared starting from K[(3,4,5-$C_6H_2F_3$)BF_3] (400 mg, 1.68 mmol) in CH_2Cl_2 (3 mL) and BF_3·OEt_2 (0.14 mL, 1.12 mmol) in CH_2Cl_2 (1 mL). The obtained CH_2Cl_2 solution containing **4-B** was analyzed by ^{11}B and ^{19}F NMR spectroscopy:

^{11}B NMR (160.46 MHz, CD_2Cl_2, 298 K; Figure S7): δ_B/ppm = 12.14 (br s).

^{19}F NMR (470.59 MHz, CD_2Cl_2, 298 K; Figure S8): δ_F/ppm = −129.16 (br s, 2F, BF_2), −136.35 (br s, 2F, *m*-F), −159.52 (m, 1F, *p*-F).

2.5. Reaction of K[(2,6-$C_6H_3F_2$)BF_3] with BF_3·OEt_2 to obtain **5-B**

By following the procedure described for the generation of **1-B**, a CH_2Cl_2 solution of **5-B** was prepared starting from K[(2,6-$C_6H_3F_2$)BF_3] (750 mg, 3.41 mmol) in CH_2Cl_2 (2.5 mL) and BF_3·OEt_2 (0.28 mL, 2.27 mmol) in CH_2Cl_2 (1.5 mL). The obtained CH_2Cl_2 solution containing **5-B** was analyzed by ^{11}B and ^{19}F NMR spectroscopy:

^{11}B NMR (160.46 MHz, CD_2Cl_2, 298 K; Figure S9): δ_B/ppm = 8.88 (br s).

^{19}F NMR (470.59 MHz, CD_2Cl_2, 298 K; Figure S10): δ_F/ppm = −104.29 (br s, 2F, *o*-F), −128.55 (br s, 2F, BF_2).

2.6. Reaction of K[(3,5-$C_6H_3F_2$)BF_3] with BF_3·OEt_2 to obtain **6-B**

By following the procedure described for the generation of **1-B**, a CH_2Cl_2 solution of **6-B** was prepared starting from K[(3,5-$C_6H_3F_2$)BF_3] (760 mg, 3.45 mmol) in CH_2Cl_2 (4 mL) and BF_3·OEt_2 (0.28 mL, 2.30 mmol) in CH_2Cl_2 (1 mL). The obtained CH_2Cl_2 solution containing **6-B** was analyzed by ^{11}B and ^{19}F NMR spectroscopy:

^{11}B NMR (160.46 MHz, CD_2Cl_2, 298 K; Figure S11): δ_B/ppm = 12.99 (br s).

^{19}F NMR (470.59 MHz, CD_2Cl_2, 298 K; Figure S12): δ_F/ppm = −110.78 (m, 2F, *m*-F), −125.91 (br s, 2F, BF_2).

2.7. Reaction of K[(2-C_6H_4F)BF_3] with BF_3·OEt_2 to obtain **7-B**

By following the procedure described for the generation of **1-B**, a CH_2Cl_2 solution of **7-B** was prepared starting from K[(2-C_6H_4F)BF_3] (250 mg, 1.24 mmol) in CH_2Cl_2 (2 mL) and BF_3·OEt_2 (0.10 mL, 0.825 mmol) in CH_2Cl_2 (1 mL). The obtained CH_2Cl_2 solution containing **7-B** was analyzed by ^{11}B and ^{19}F NMR spectroscopy:

^{11}B NMR (160.46 MHz, CD_2Cl_2, 298 K; Figure S13): δ_B/ppm = 15.21 (br s).

^{19}F NMR (470.59 MHz, CD_2Cl_2, 298 K; Figure S14): δ_F/ppm = −105.45 (br s, 1F, *o*-F), −115.77 (br s, 2F, BF_2).

2.8. Reaction of K[(3-C_6H_4F)BF_3] with BF_3·OEt_2 to obtain **8-B**

By following the procedure described for the generation of **1-B**, a CH_2Cl_2 solution of **8-B** was prepared starting from K[(3-C_6H_4F)BF_3] (640 mg, 3.17 mmol) in CH_2Cl_2 (2.5 mL) and BF_3·OEt_2 (0.26 mL, 2.11 mmol) in CH_2Cl_2 (1 mL). The obtained CH_2Cl_2 solution containing **8-B** was analyzed by ^{11}B and ^{19}F NMR spectroscopy:

^{11}B NMR (160.46 MHz, CD_2Cl_2, 298 K; Figure S15): δ_B/ppm = 15.08 (br s).

^{19}F NMR (470.59 MHz, CD_2Cl_2, 298 K; Figure S16): δ_F/ppm = −114.17 (m, 1F, *m*-F), −120.97 (br s, 2F, BF_2).

2.9. Reaction of K[(4-C_6H_4F)BF_3] with BF_3·OEt_2 to obtain **9-B**

By following the procedure described for the generation of **1-B**, a CH_2Cl_2 solution of **9-B** was prepared starting from K[(4-C_6H_4F)BF_3] (300 mg, 1.49 mmol) in CH_2Cl_2 (2 mL) and BF_3·OEt_2 (0.12 mL, 0.990 mmol) in CH_2Cl_2 (1 mL). The obtained CH_2Cl_2 solution containing **9-B** was analyzed by ^{11}B and ^{19}F NMR spectroscopy:

^{11}B NMR (160.46 MHz, CD_2Cl_2, 298 K; Figure S17): δ_B/ppm = 20.57 (br s).

^{19}F NMR (470.59 MHz, CD_2Cl_2, 298 K; Figure S18): δ_F/ppm = −105.34 (br s, 2F, BF_2), −106.64 (br s, 1F, *p*-F).

2.10. Reaction of K[(C_6H_5)BF_3] with BF_3·OEt_2 to obtain **10-B**

By following the procedure described for the generation of **1-B**, a CH_2Cl_2 solution of **10-B** was prepared starting from K[(C_6H_5)BF_3] (440 mg, 2.39 mmol) in CH_2Cl_2 (2 mL) and BF_3·OEt_2 (0.20 mL, 1.59 mmol) in CH_2Cl_2 (1 mL). The obtained CH_2Cl_2 solution containing **10-B** was analyzed by ^{11}B and ^{19}F NMR spectroscopy:

^{11}B NMR (160.46 MHz, CD_2Cl_2, 298 K; Figure S19): δ_B/ppm = 22.95 (m).

^{19}F NMR (470.59 MHz, CD_2Cl_2, 298 K; Figure S20): δ_F/ppm = −98.85 (br s, 2F, BF_2).

2.11. Synthetic use of aryldifluoroborane solutions

The dichloromethane solutions of aryldifluoroboranes, generated as described in this section, can be used directly for the synthesis of arylxenonium(II) tetrafluoroborates. Based on further transformations, the practical yield obtained of these reactions was determined to be approximately 60%. Accordingly, the experimental procedures outlined in Section 3.1 were conducted on this basis. The presence of a signal at ca. 0 ppm in some of the ^{11}B NMR spectra corresponds to fluoroborates arising from adventitious traces of water, and have no influence in the subsequent transformation to the arylxenonium(II) salt.

3. Synthesis of arylxenonium(II) tetrafluoroborates

3.1. Synthesis of $[RXe][BF_4]$ salts (R = fluorinated aryl group) from CH_2Cl_2 solutions of RBF_2 species

3.1.1. Synthesis of $[(2,4,6\text{-}C_6H_2F_3)Xe][BF_4]$ (**3-Xe**)

A freshly prepared solution of **3-B** (1.55 mmol) was added dropwise to a saturated solution of XeF_2 (262 mg, 1.55 mmol) in CH_2Cl_2 (6 mL) at −40 °C. After stirring for 2 h at −40 °C, a white suspension was formed, which was kept at −86 °C overnight to allow the solid to be deposited. The yellow supernatant was then filtered off before washing with cold CH_2Cl_2 (2 × 0.5 mL). After drying the white material under vacuum at low temperature, compound **3-Xe** was obtained as a white solid (241 mg, 0.690 mmol, 45% yield). The spectroscopic data are in agreement with those previously reported.[6]

^{1}H NMR (300.13 MHz, CD_3CN, 298 K; Figure S25): δ_H/ppm = 7.38 (m, 2H, *m*-H).

^{19}F NMR (282.40 MHz, CD_3CN, 298 K; Figure S26): δ_F/ppm = −97.56 (m, 1F, *p*-F), −97.76 (m, 2F, $^3J(^{19}F_o, ^{129}Xe)$ = 57.2 Hz, *o*-F), −151.26 (br s, 4F, BF_4).

3.1.2. Synthesis of $[(3,4,5\text{-}C_6H_2F_3)Xe][BF_4]$ (**4-Xe**)

By following the procedure described for the synthesis of **3-Xe**, compound **4-Xe** was prepared starting from a solution of XeF_2 (114 mg, 0.672 mmol) in CH_2Cl_2 (2.5 mL) and a freshly prepared solution of **4-B** (0.672 mmol). The crude product was washed with cold CH_2Cl_2 (2 × 0.5 mL) and then dried under vacuum. Compound **4-Xe** was obtained as a white solid (98.5 mg, 0.282 mmol, 42% yield). The spectroscopic data are in agreement with those previously reported.[7]

^{1}H NMR (300.13 MHz, CD_3CN, 233 K; Figure S27): δ_H/ppm = 8.01 (t, 2H, $^3J(^1H_o, ^{19}F_m)$ = 5.7 Hz, $^3J(^1H_o, ^{129}Xe)$ = 22.3 Hz, *o*-H).

^{19}F NMR (282.40 MHz, CD_3CN, 233 K; Figure S28): δ_F/ppm = −127.12 (m, 2F, *m*-F), −150.89 (br s, 4F, BF_4), −151.38 (tt, 1F, $^3J(^{19}F_m, ^{19}F_p)$ = 19.6 Hz, $^4J(^1H_o, ^{19}F_p)$ = 5.7 Hz, *p*-F).

3.1.3. Synthesis of [(2,6-$C_6H_3F_2$)Xe][BF_4] (**5-Xe**)

By following the procedure described for the synthesis of **3-Xe**, compound **5-Xe** was prepared starting from a solution of XeF_2 (231 mg, 1.36 mmol) in CH_2Cl_2 (10 mL) and a freshly prepared solution of **5-B** (1.36 mmol). The crude product was washed with cold CH_2Cl_2 (2 × 1 mL) and then dried under vacuum. Compound **5-Xe** was obtained as a white solid (217 mg, 0.655 mmol, 48% yield). The spectroscopic data are in agreement with those previously reported.[8]

^{1}H NMR (300.13 MHz, CD_3CN, 298 K; Figure S29): δ_H/ppm = 7.89 (tt, 1H, $^3J(^1H_m, ^{19}F_o)$ = 6.2 Hz, $^3J(^1H_m, ^1H_p)$ = 8.6 Hz, *p*-H), 7.45 (m, 2H, *m*-H).

^{19}F NMR (282.40 MHz, CD_3CN, 298 K; Figure S30): δ_F/ppm = −100.80 (m, 2F, $^3J(^{19}F_o, ^{129}Xe)$ = 52.9 Hz, *o*-F), −151.32 (br s, 4F, BF_4).

3.1.4. Synthesis of [(3,5-$C_6H_3F_2$)Xe][BF_4] (**6-Xe**)

By following the procedure described for the synthesis of **3-Xe**, compound **6-Xe** was prepared starting from a solution of XeF_2 (234 mg, 1.38 mmol) in CH_2Cl_2 (10 mL) and a freshly prepared solution of **6-B** (1.38 mmol) The crude product was washed with cold CH_2Cl_2 (2 × 0.5 mL) and then dried under vacuum. Compound **6-Xe** was obtained as a white solid (232 mg, 0.701 mmol, 51% yield). The spectroscopic data are in agreement with those previously reported.[7]

^{1}H NMR (300.13 MHz, CD_3CN, 233 K; Figure S31): δ_H/ppm = 7.85 (dd, sh, 2H, $^3J(^1H_o, ^{19}F_m)$ = 5.2 Hz, $^4J(^1H_o, ^1H_p)$ = 2.2 Hz, $^3J(^1H_o, ^{129}Xe)$ = unresolved signal, *o*-H), 7.54 (tt, 1H, t, 2H, $^3J(^1H_p, ^{19}F_m)$ = 9.0 Hz, $^4J(^1H_o, ^1H_p)$ = 2.2 Hz, *p*-H).

^{19}F NMR (282.40 MHz, CD_3CN, 233 K; Figure S32): δ_F/ppm = −102.55 (m, 2F, *m*-F), −150.89 (br s, 4F, BF_4).

3.1.5. Synthesis of [(2-C_6H_4F)Xe][BF_4] (**7-Xe**)

By following the procedure described for the synthesis of **3-Xe**, compound **7-Xe** was prepared starting from a solution of XeF_2 (83.8 mg, 0.495 mmol) in CH_2Cl_2 (2 mL) and a freshly prepared solution of **7-B** (0.495 mmol). The crude product was washed with cold CH_2Cl_2 (2 × 0.5 mL) and then dried under vacuum. Compound **7-Xe** was obtained

as a white solid (148 mg, 0.473 mmol, 95% yield). The spectroscopic data are in agreement with those previously reported.[8]

^{1}H NMR (300.13 MHz, CD_3CN, 298 K; Figure S33): δ_H/ppm = 8.19 (m, $^3J(^1H_o, ^{129}Xe)$ = unresolved signal, 1H, *o*-H), 7.84 (m, 1H, m_5-H), 7.65 (m, 1H, *p*-H), 7.43 (m, 1H, m_3-H).

^{19}F NMR (282.40 MHz, CD_3CN, 298 K; Figure S34): δ_F/ppm = −101.50 (m, 1H, $^3J(^{19}F_o, ^{129}Xe)$ = 48.5 Hz, *o*-H), −151.45 (br s, 4F, BF_4).

3.1.6. Synthesis of [(3-C_6H_4F)Xe][BF_4] (**8-Xe**)

By following the procedure described for the synthesis of **3-Xe**, compound **8-Xe** was prepared starting from a solution of XeF_2 (215 mg, 1.27 mmol) in CH_2Cl_2 (5 mL) and a freshly prepared solution of **8-B** (1.27 mmol). The crude product was washed with cold CH_2Cl_2 (3 × 1 mL) and then dried under vacuum. Compound **8-Xe** was obtained as a white solid (340 mg, 1.086 mmol, 86% yield). The spectroscopic data are in agreement with those previously reported.[9]

^{1}H NMR (300.13 MHz, CD_3CN, 233 K; Figure S35): δ_H/ppm = 7.78−7.52 (m, 4H, aromatic H).

^{19}F NMR (282.40 MHz, CD_3CN, 233 K; Figure S36): δ_F/ppm = −109.64 (m, 1F, *m*-F), −150.40 (br s, 4F, BF_4).

3.1.7. Synthesis of [(4-C_6H_4F)Xe][BF_4] (**9-Xe**)

By following the procedure described for the synthesis of **3-Xe**, compound **9-Xe** was prepared starting from a solution of XeF_2 (101 mg, 0.594 mmol) in CH_2Cl_2 (2 mL) and a freshly prepared solution of **9-B** (0.594 mmol) The crude product was washed with cold CH_2Cl_2 (2 × 1 mL) and then dried under vacuum. Compound **9-Xe** was obtained as a white solid (143 mg, 0.457 mmol, 77% yield). The spectroscopic data are in agreement with those previously reported.[8,9]

^{1}H NMR (300.13 MHz, CD_3CN, 233 K; Figure S37): δ_H/ppm = 7.86 (m, 2H, *o*-H), 7.41 (m, 2H, *m*-H).

^{19}F NMR (282.40 MHz, CD_3CN, 233 K; Figure S38): δ_F/ppm = −103.25 (m, 1F, *p*-F), −151.02 (br s, 4F, BF_4).

3.2. One-pot synthesis of [RXe][BF_4] salts (R = fluorinated aryl group)

3.2.1. Synthesis of [(C_6F_5)Xe][BF_4] (**1-Xe**)

B(C_6F_5)$_3$ (1.00 g, 1.95 mmol) was suspended in CH_2Cl_2 (25 mL) and BF_3·OEt_2 (0.48 mL, 3.91 mmol) was then slowly added before cooling the borane mixture to −40°C. XeF_2 (992 mg, 5.86 mmol) was dissolved in CH_2Cl_2 (25 mL) at −40°C and then added dropwise to the borane solution. The reaction mixture was left stirring for 4 h at −78°C and then the white solid was allowed to settle at −86°C. The yellow supernatant was filtered off before washing with cold CH_2Cl_2 (2 × 4 mL). After drying under vacuum, compound **1-Xe** was obtained as a white solid (0.998 mg, 2.59 mmol, 44% yield). The spectroscopic data are in agreement with those previously reported.[10]

^{19}F NMR (282.40 MHz, CD_3CN, 298 K; Figure S21): δ_F/ppm = −126.42 (m, 2F, $^3J(^{19}F_o, {}^{129}Xe)$ = 68.4 Hz, *o*-F), −142.76 (tt, 1F, $^3J(^{19}F_m, {}^{19}F_p)$ = 20.0 Hz, $^4J(^{19}F_o, {}^{19}F_p)$ = 5.6 Hz, *p*-F), −151.62 (br s, 4F, BF_4), −155.69 (m, 2F, *m*-F).

3.2.2. Synthesis of [(2,3,5,6-C_6HF_4)Xe][BF_4] (**2-Xe**)

By following the procedure described for the synthesis of **1-Xe**, [(2,3,5,6-C_6HF_4)Xe][BF_4] (**2-Xe**) was prepared starting from a suspension of B(2,3,5,6-C_6HF_4)$_3$ (100 mg, 0.218 mmol) in CH_2Cl_2 (2 mL), BF_3·OEt_2 (0.05 mL, 0.437 mmol) and a solution of XeF_2 (111 mg, 0.655 mmol) in CH_2Cl_2 (2.5 mL) at −40°C. After washing the crude product with cold CH_2Cl_2 (2 × 1 mL) and then dried under vacuum, compound **2-Xe** was obtained as a white solid (69.1 mg, 0.188 mmol, 29% yield).

^{1}H NMR (300.13 MHz, CD_3CN, 298 K; Figure S22): δ_H/ppm = 8.00 (tt, 1H, $^3J(^1H_p, {}^{19}F_m)$ = 9.88 Hz, $^4J(^1H_p, {}^{19}F_o)$ = 7.41 Hz, *p*-H).

^{19}F NMR (282.40 MHz, CD_3CN, 298 K; Figure S23): δ_F/ppm = −127.53 (m, 2F, $^3J(^{19}F_o, {}^{129}Xe)$ = 63.4 Hz, *o*-F), −133.53 (m, 2F, *m*-F), −151.18 (br s, 4F, BF_4).

^{129}Xe NMR (83.47 MHz, CD_3CN, 298 K; Figure S24): δ_{Xe}/ppm = −3805.7 (t, 1Xe, $^3J(^{19}F_o, {}^{129}Xe)$ = 63.4 Hz, Xe).

4. NMR Spectra

4.1. NMR spectra of the reaction of K[RBF_3] salts (R = aryl group) with BF_3·OEt_2

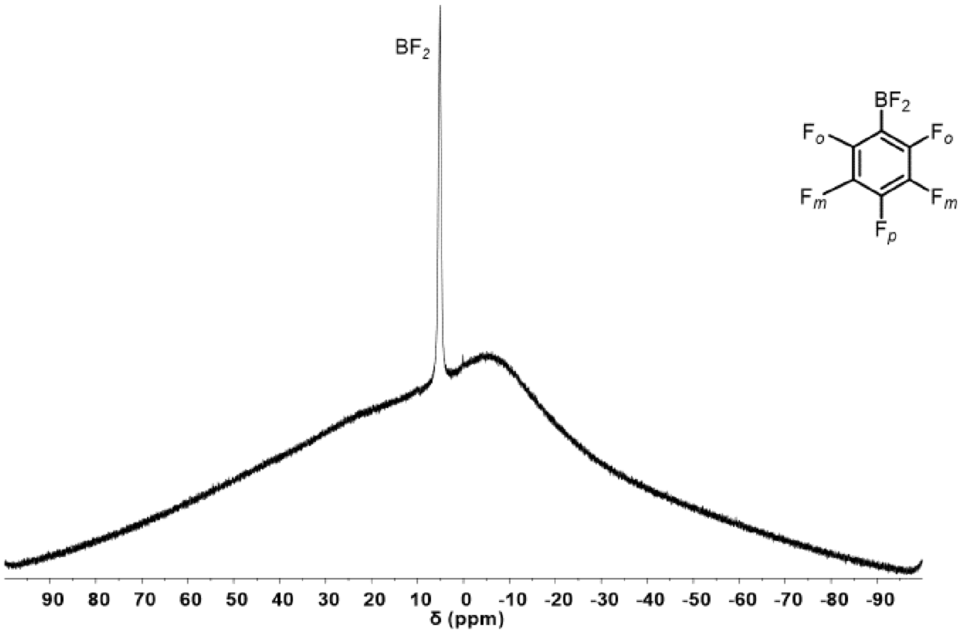

Figure S1. ^{11}B NMR spectrum (160.46 MHz, CD_2Cl_2, 298 K) of the reaction of K[(C_6F_5)BF_3] with BF_3·OEt_2.

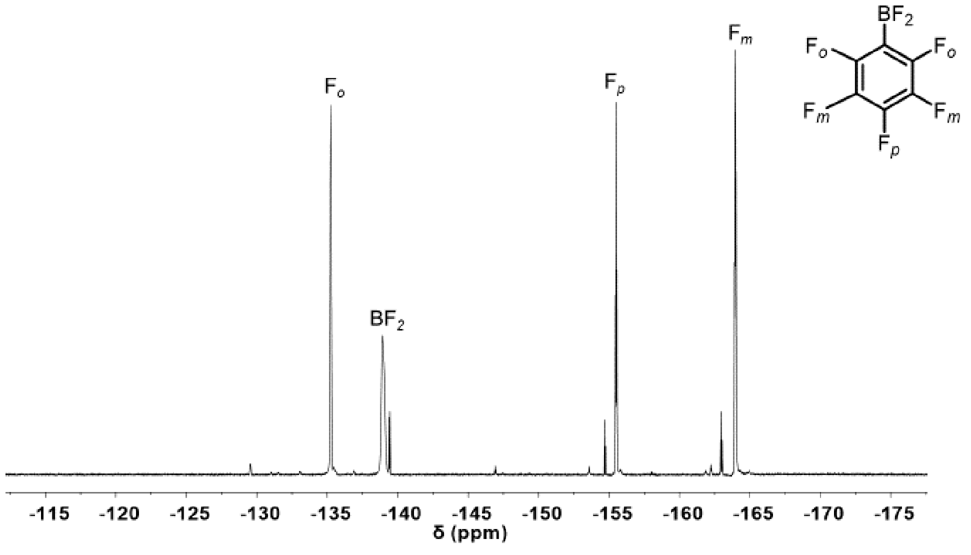

Figure S2. ^{19}F NMR spectrum (470.59 MHz, CD_2Cl_2, 298 K) of the reaction of K[(C_6F_5)BF_3] with BF_3·OEt_2. Additional signals correspond to hydrolysis products due to adventitious traces of water.

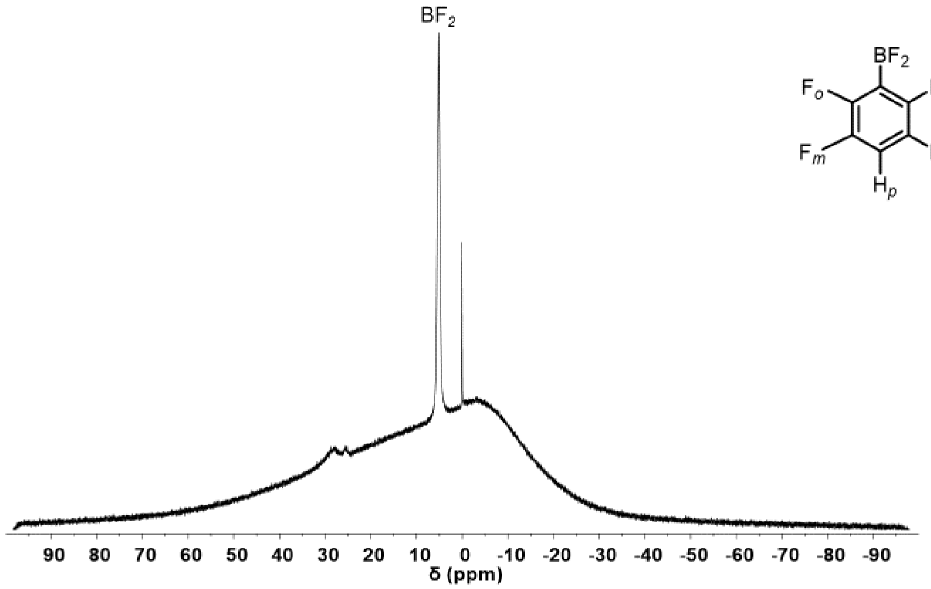

Figure S3. ^{11}B NMR spectrum (160.46 MHz, CD_2Cl_2, 298 K) of the reaction of $K[(2,3,5,6\text{-}C_6HF_4)BF_3]$ with $BF_3{\cdot}OEt_2$.

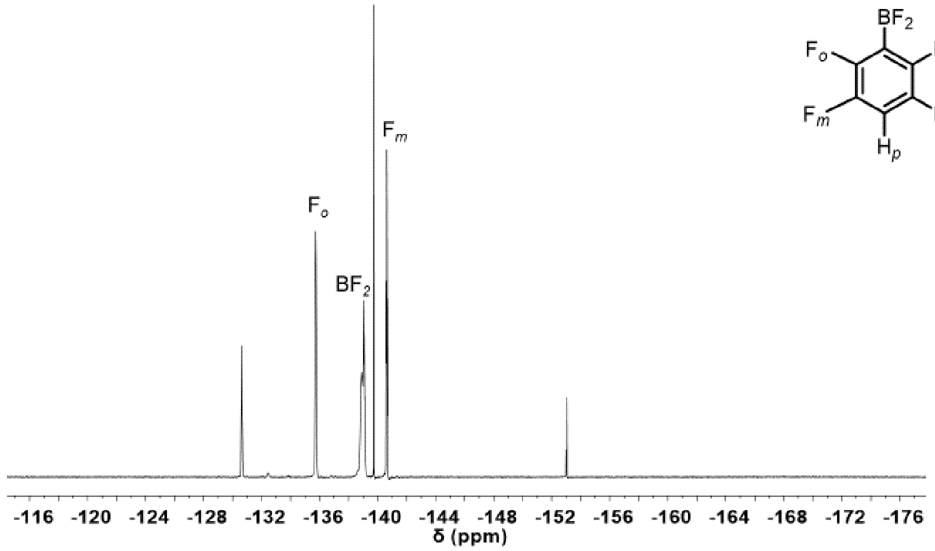

Figure S4. ^{19}F NMR spectrum (470.59 MHz, CD_2Cl_2, 298 K) of the reaction of $K[(2,3,5,6\text{-}C_6HF_4)BF_3]$ with $BF_3{\cdot}OEt_2$. Additional signals correspond to hydrolysis products due to adventitious traces of water.

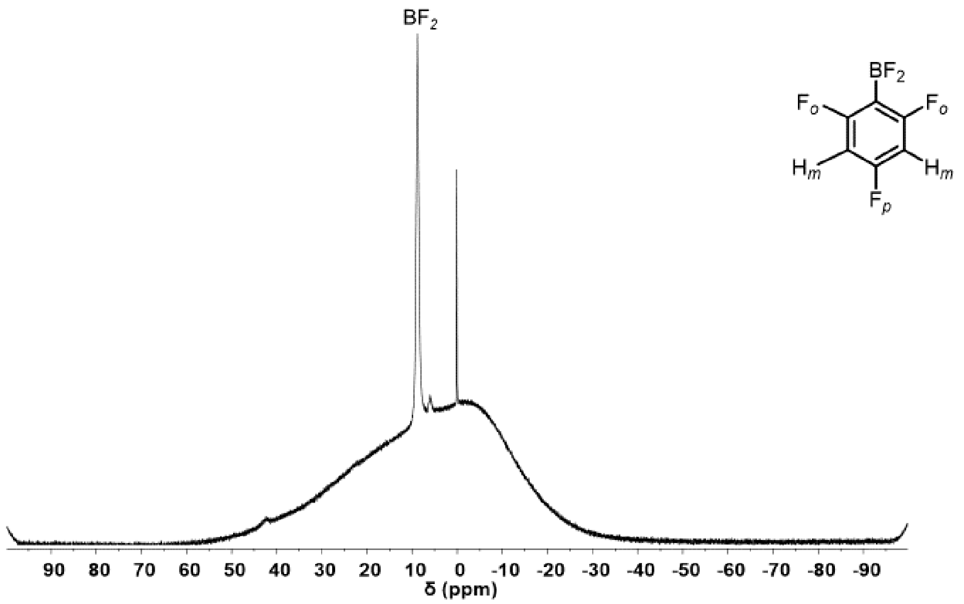

Figure S5. ^{11}B NMR spectrum (160.46 MHz, CD_2Cl_2, 298 K) of the reaction of $K[(2,4,6\text{-}C_6H_2F_3)BF_3]$ with $BF_3{\cdot}OEt_2$.

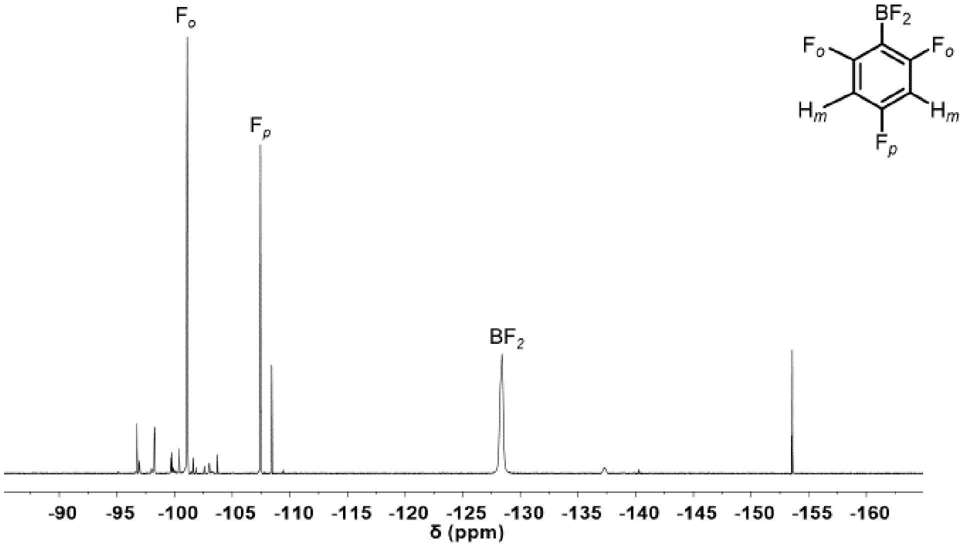

Figure S6. ^{19}F NMR spectrum (470.59 MHz, CD_2Cl_2, 298 K) of the reaction of $K[(2,4,6\text{-}C_6H_2F_3)BF_3]$ with $BF_3{\cdot}OEt_2$. Additional signals correspond to hydrolysis products due to adventitious traces of water, with no impact in the subsequent reaction to prepare **3-Xe**.

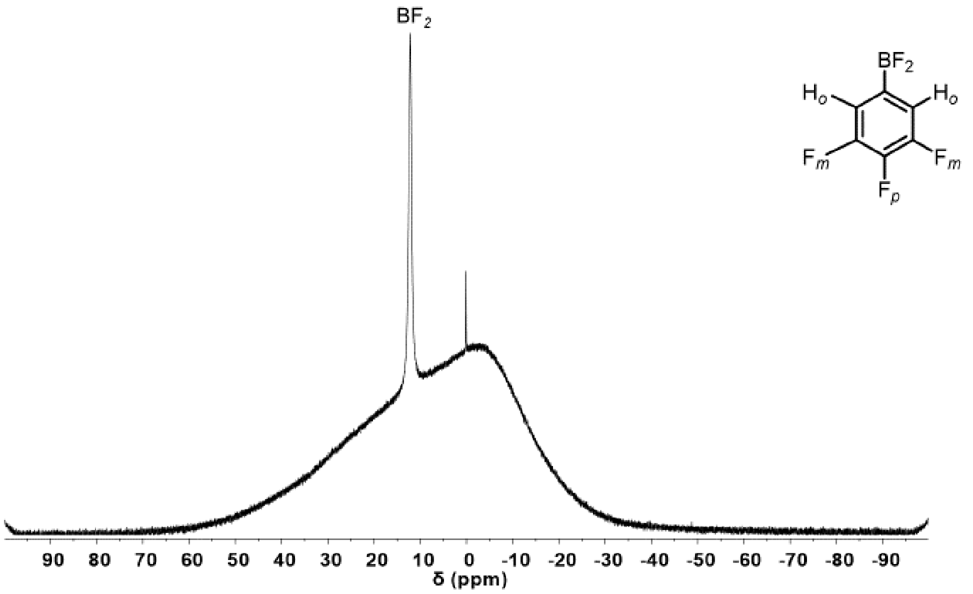

Figure S7. ^{11}B NMR spectrum (160.46 MHz, CD_2Cl_2, 298 K) of the reaction of K[(3,4,5-$C_6H_2F_3$)BF_3] with $BF_3 \cdot OEt_2$.

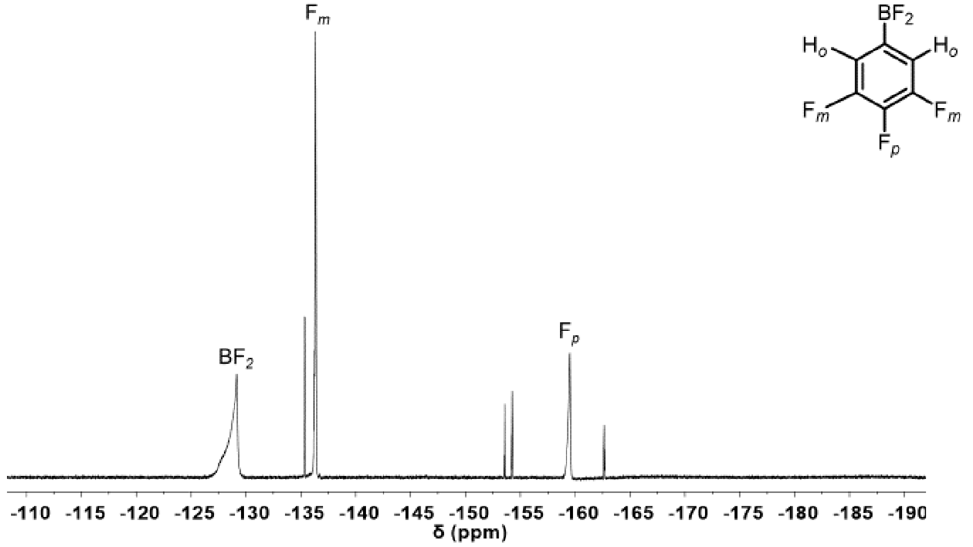

Figure S8. ^{19}F NMR spectrum (470.59 MHz, CD_2Cl_2, 298 K) of the reaction of K[(3,4,5-$C_6H_2F_3$)BF_3] with $BF_3 \cdot OEt_2$. Additional signals correspond to hydrolysis products due to adventitious traces of water, with no impact in the subsequent reaction to prepare **4-Xe**.

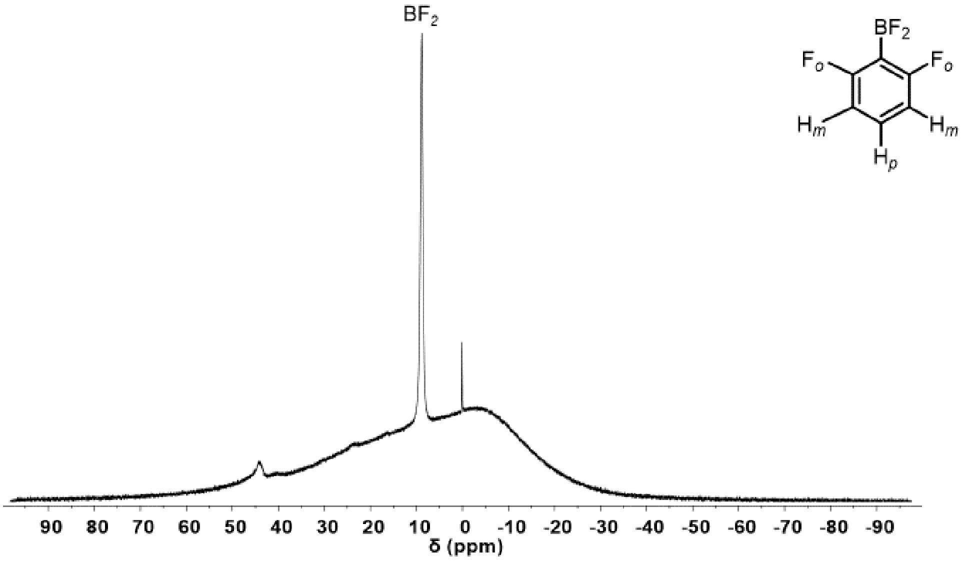

Figure S9. ^{11}B NMR spectrum (160.46 MHz, CD_2Cl_2, 298 K) of the reaction of K[(2,6-$C_6H_3F_2$)BF_3] with $BF_3 \cdot OEt_2$.

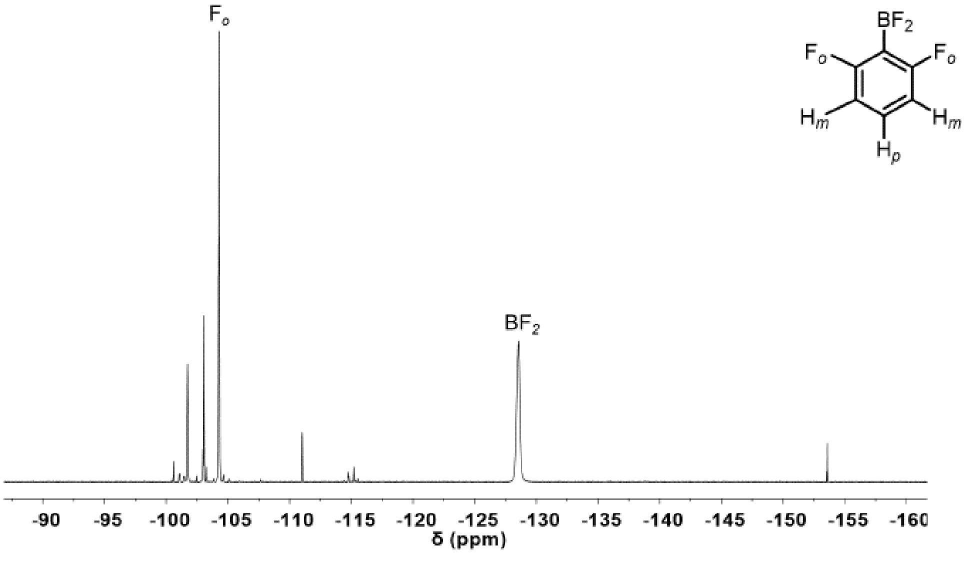

Figure S10. ^{19}F NMR spectrum (470.59 MHz, CD_2Cl_2, 298 K) of the reaction of K[(2,6-$C_6H_3F_2$)BF_3] with $BF_3 \cdot OEt_2$. Additional signals correspond to hydrolysis products due to adventitious traces of water, with no impact in the subsequent reaction to prepare **5-Xe**.

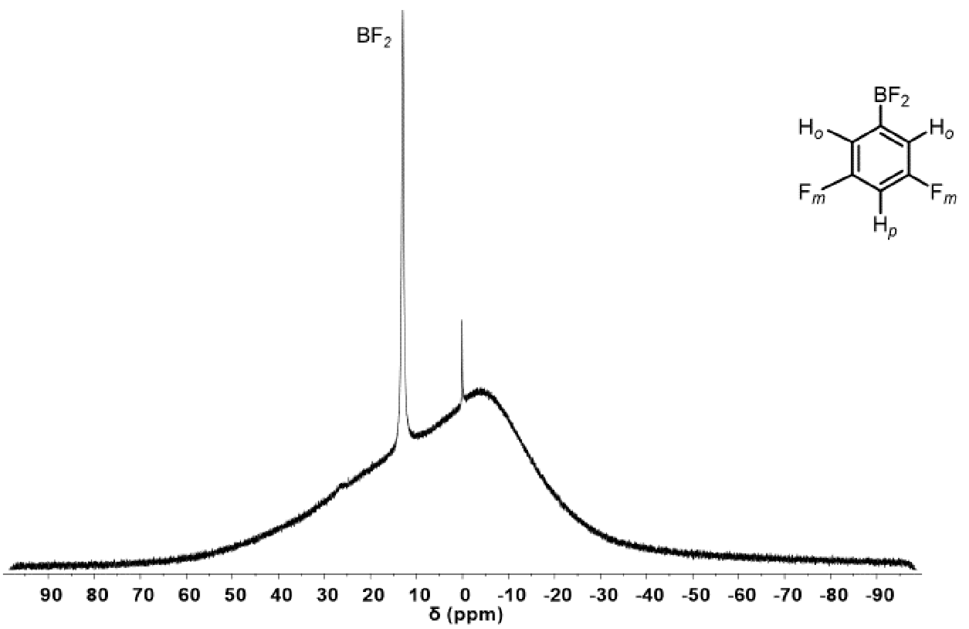

Figure S11. ^{11}B NMR spectrum (160.46 MHz, CD_2Cl_2, 298 K) of the reaction of $K[(3,5\text{-}C_6H_3F_2)BF_3]$ with $BF_3{\cdot}OEt_2$.

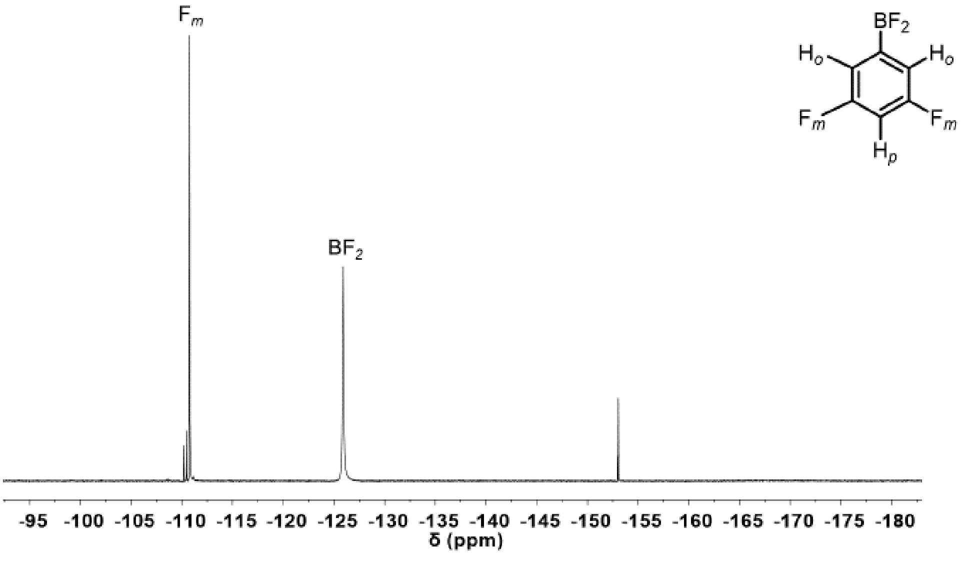

Figure S12. ^{19}F NMR spectrum (470.59 MHz, CD_2Cl_2, 298 K) of the reaction of $K[(3,5\text{-}C_6H_3F_2)BF_3]$ with $BF_3{\cdot}OEt_2$.

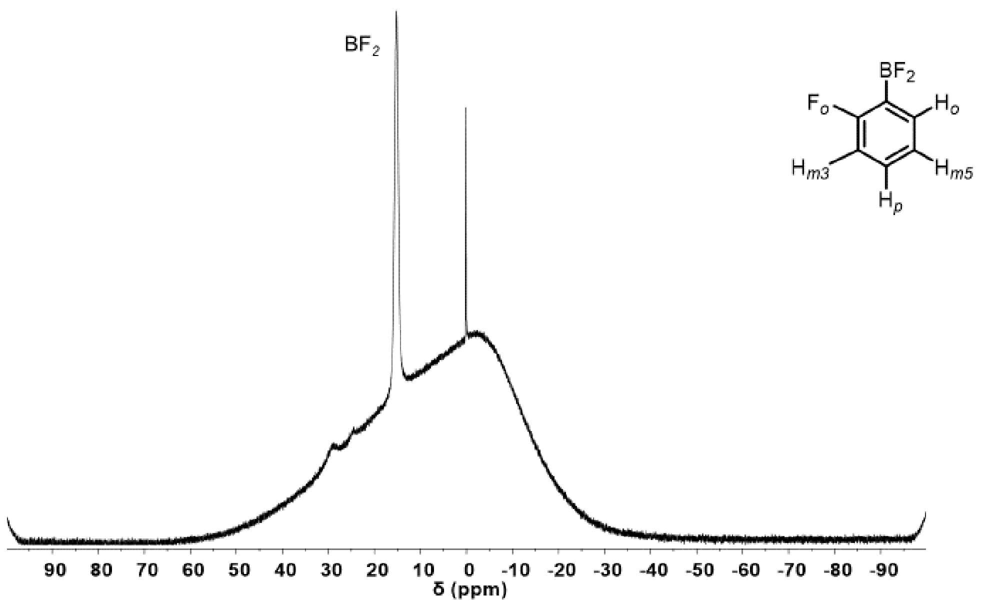

Figure S13. ^{11}B NMR spectrum (160.46 MHz, CD_2Cl_2, 298 K) of the reaction of K[(2-C_6H_4F)BF_3] with BF_3·OEt_2.

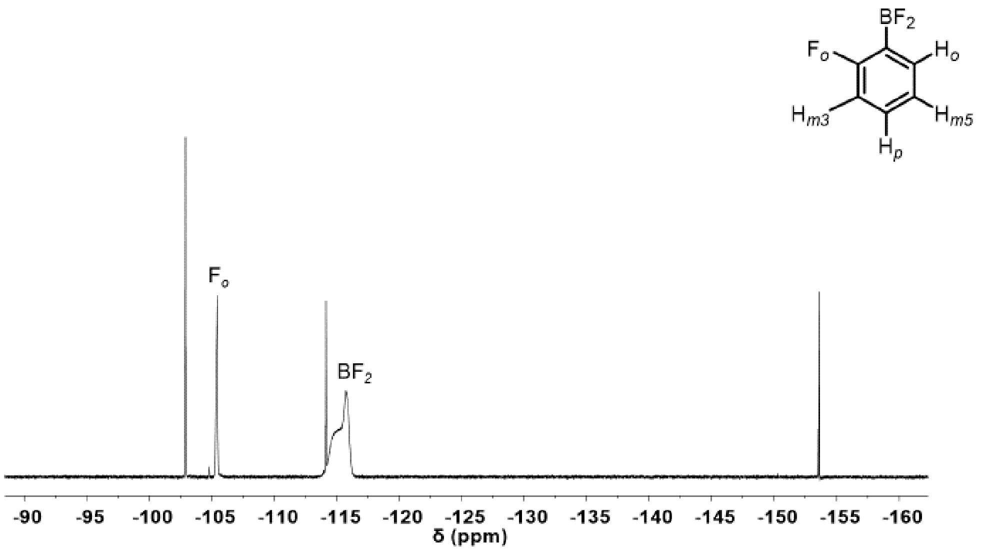

Figure S14. ^{19}F NMR spectrum (470.59 MHz, CD_2Cl_2, 298 K) of the reaction of K[(2-C_6H_4F)BF_3] with BF_3·OEt_2. Additional signals correspond to hydrolysis products due to adventitious traces of water, with no impact in the subsequent reaction to prepare **7-Xe**.

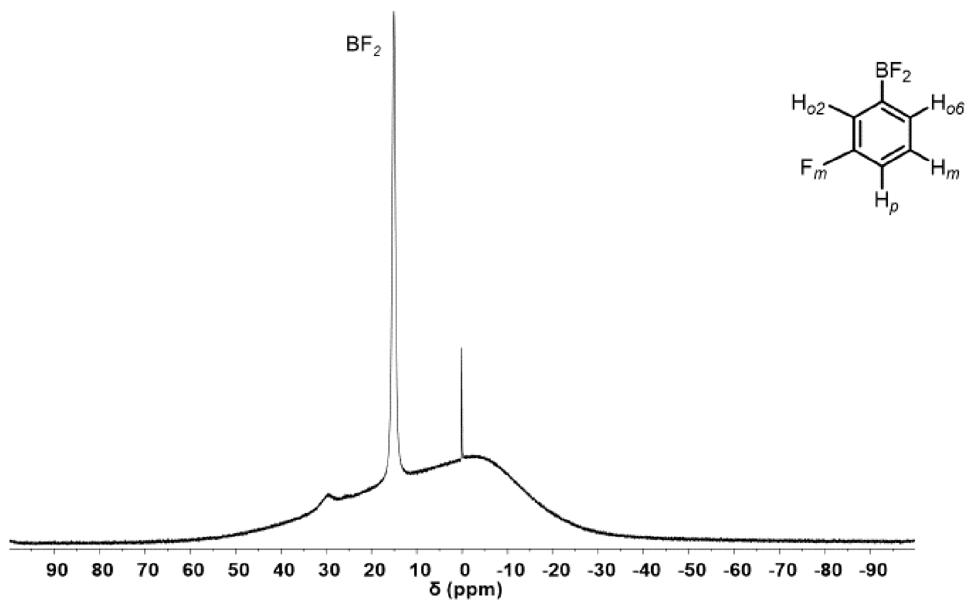

Figure S15. ^{11}B NMR spectrum (160.46 MHz, CD_2Cl_2, 298 K) of the reaction of K[(3-C_6H_4F)BF_3] with $BF_3\cdot OEt_2$.

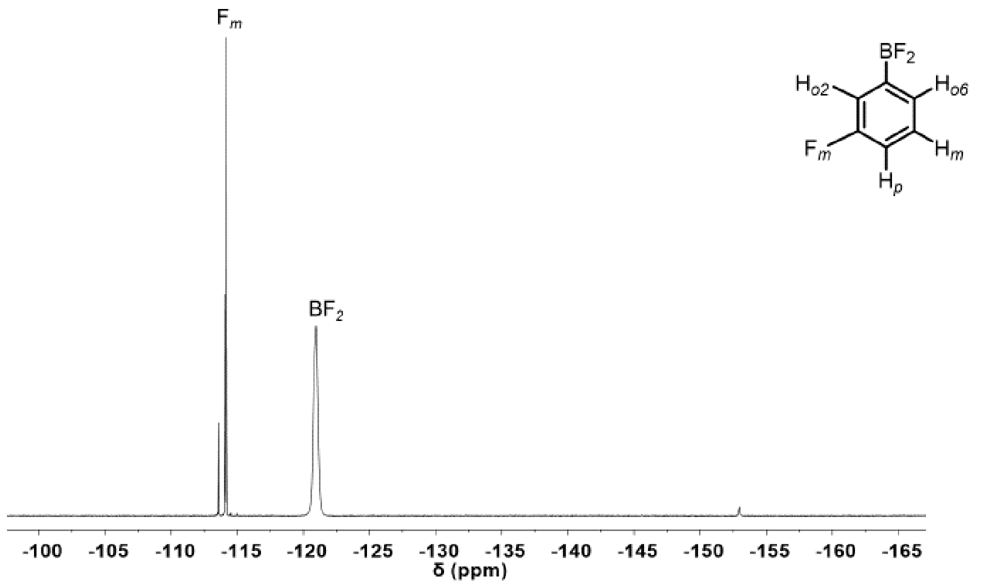

Figure S16. ^{19}F NMR spectrum (470.59 MHz, CD_2Cl_2, 298 K) of the reaction of K[(3-C_6H_4F)BF_3] with $BF_3\cdot OEt_2$.

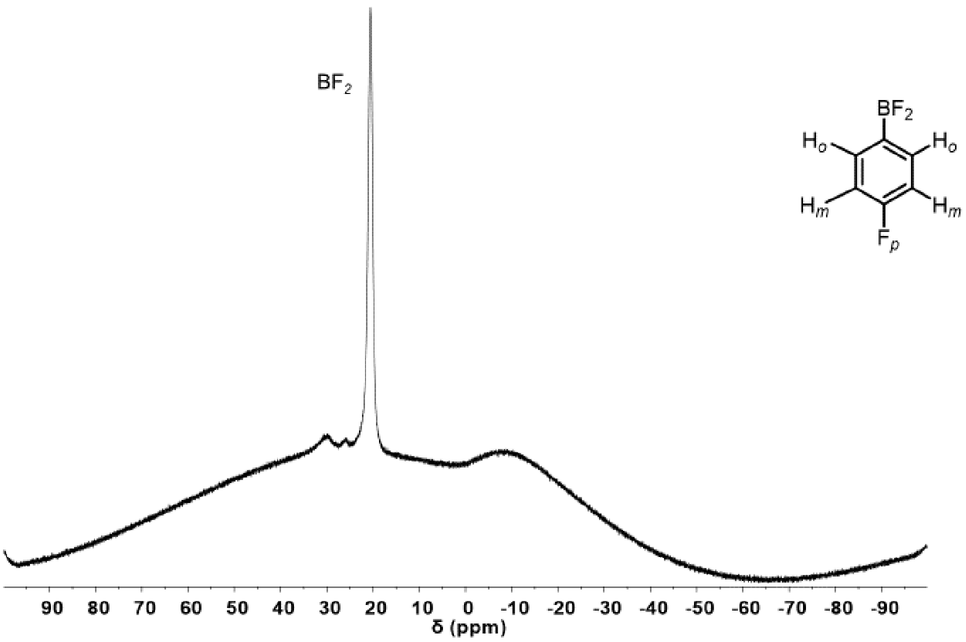

Figure S17. ^{11}B NMR spectrum (160.46 MHz, CD_2Cl_2, 298 K) of the reaction of K[(4-C_6H_4F)BF_3] with $BF_3 \cdot OEt_2$.

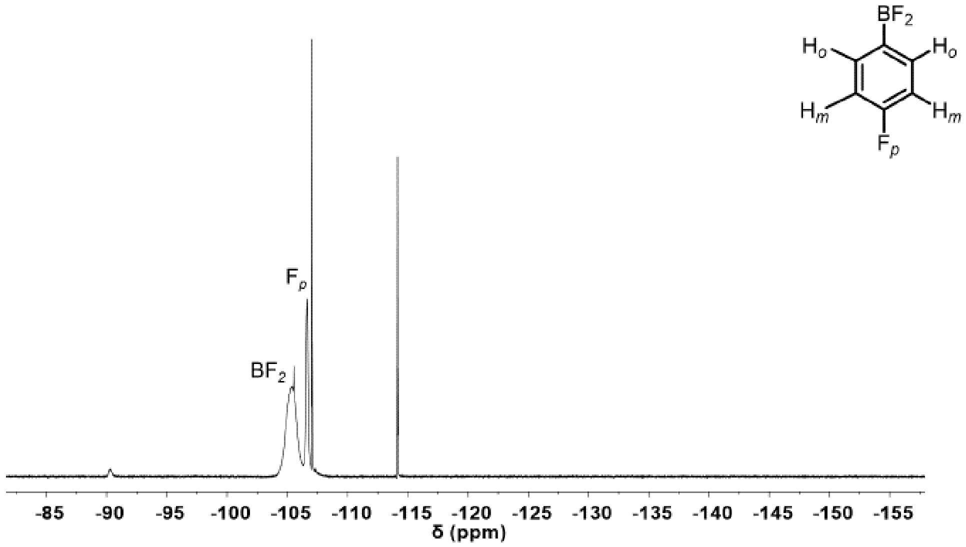

Figure S18. ^{19}F NMR spectrum (470.59 MHz, CD_2Cl_2, 298 K) of the reaction of K[(4-C_6H_4F)BF_3] with $BF_3 \cdot OEt_2$. Additional signals correspond to hydrolysis products due to adventitious traces of water, with no impact in the subsequent reaction to prepare **9-Xe**.

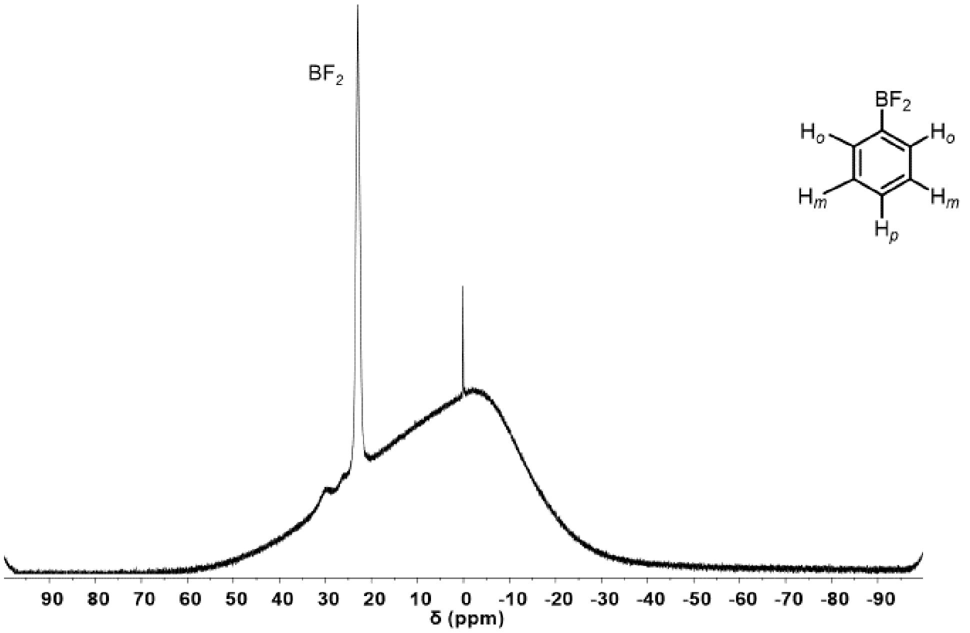

Figure S19. ^{11}B NMR spectrum (160.46 MHz, CD_2Cl_2, 298 K) of the reaction of $K[(C_6H_5)BF_3]$ with $BF_3{\cdot}OEt_2$.

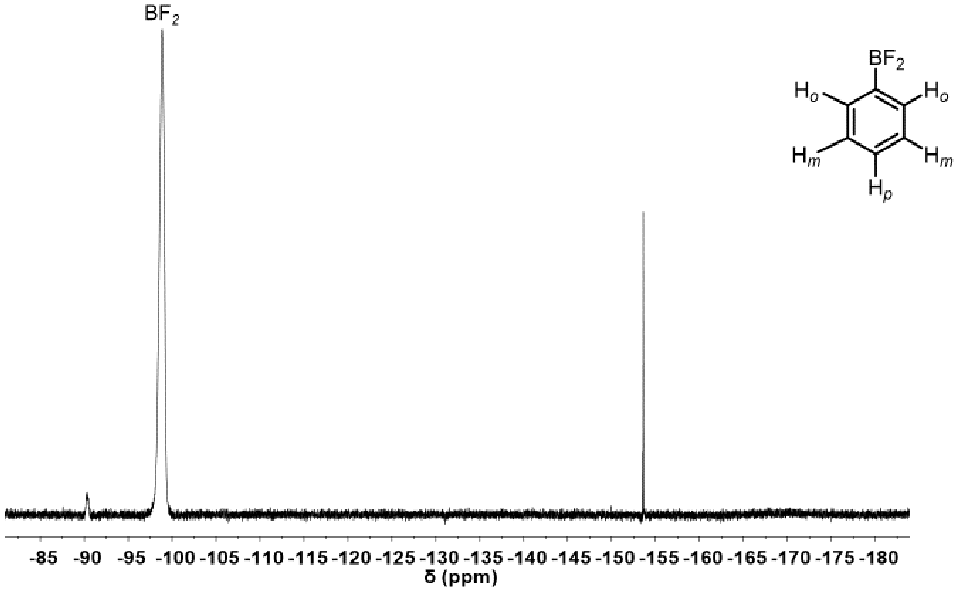

Figure S20. ^{19}F NMR spectrum (470.59 MHz, CD_2Cl_2, 298 K) of the reaction of $K[(C_6H_5)BF_3]$ with $BF_3{\cdot}OEt_2$.

4.2. NMR spectra of arylxenonium tetrafluoroborates $[RXe][BF_4]$ (R = fluorinated aryl group)

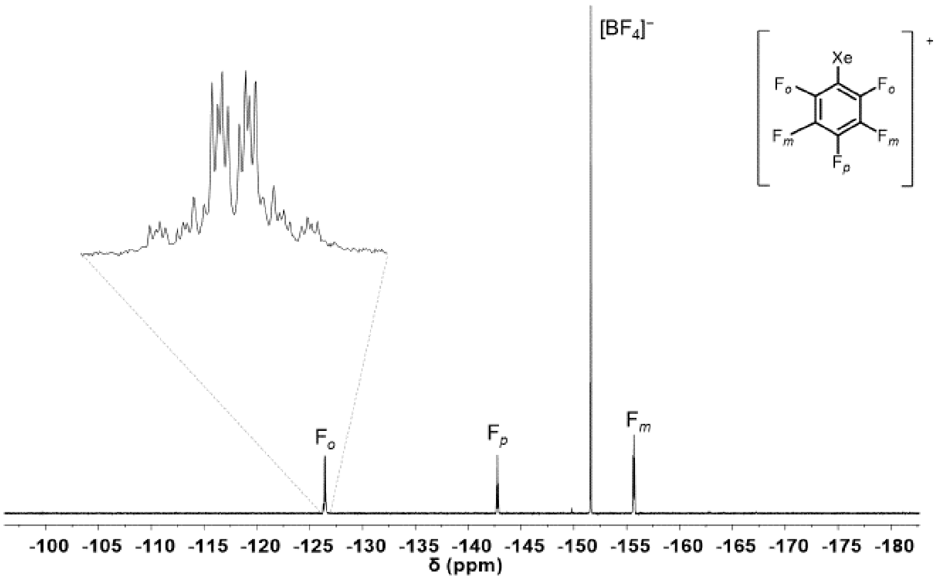

Figure S21. ^{19}F NMR spectrum (282.40 MHz, CD_3CN, 298 K) of $[(C_6F_5)Xe][BF_4]$ (**1-Xe**).

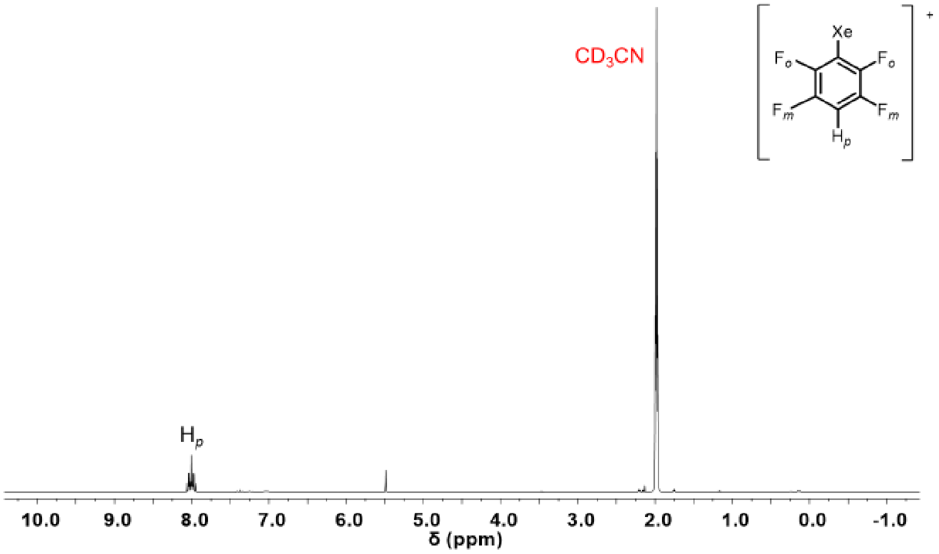

Figure S22. ^{1}H NMR spectrum (300.13 MHz, CD_3CN, 298 K) of $[(2,3,5,6\text{-}C_6HF_4)Xe][BF_4]$ (**2-Xe**).

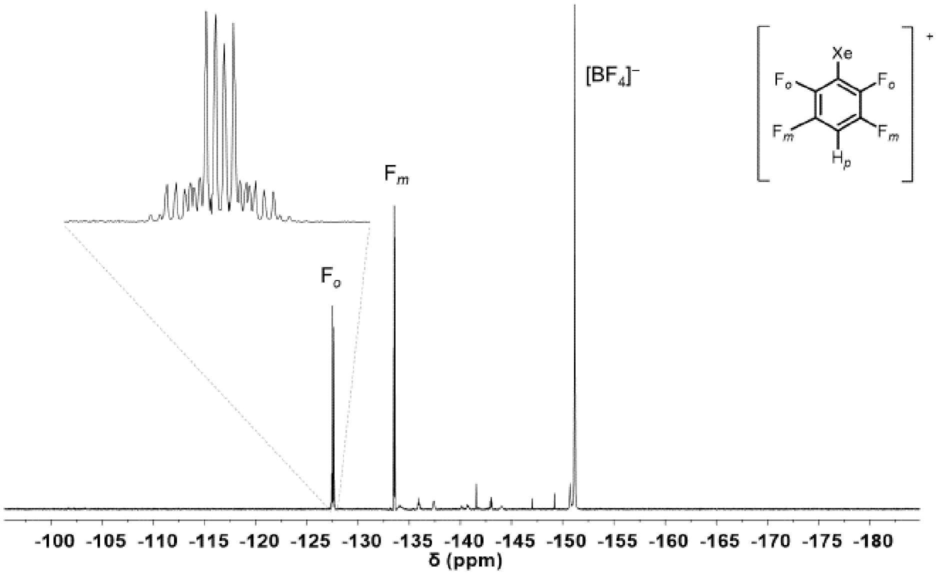

Figure S23. ^{19}F NMR spectrum (282.40 MHz, CD_3CN, 298 K) of [(2,3,5,6-(C_6HF_4)Xe][BF_4] (**2-Xe**).

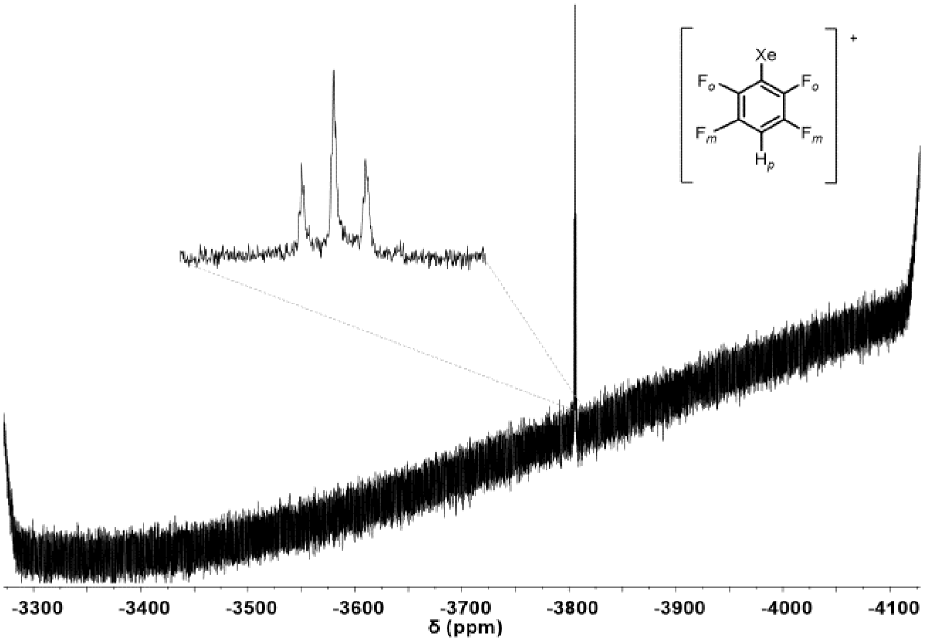

Figure S24. [129]Xe NMR spectrum (83.47 MHz, CD_3CN, 298 K) of [(2,3,5,6-C_6HF_4)Xe][BF_4] (**2-Xe**).

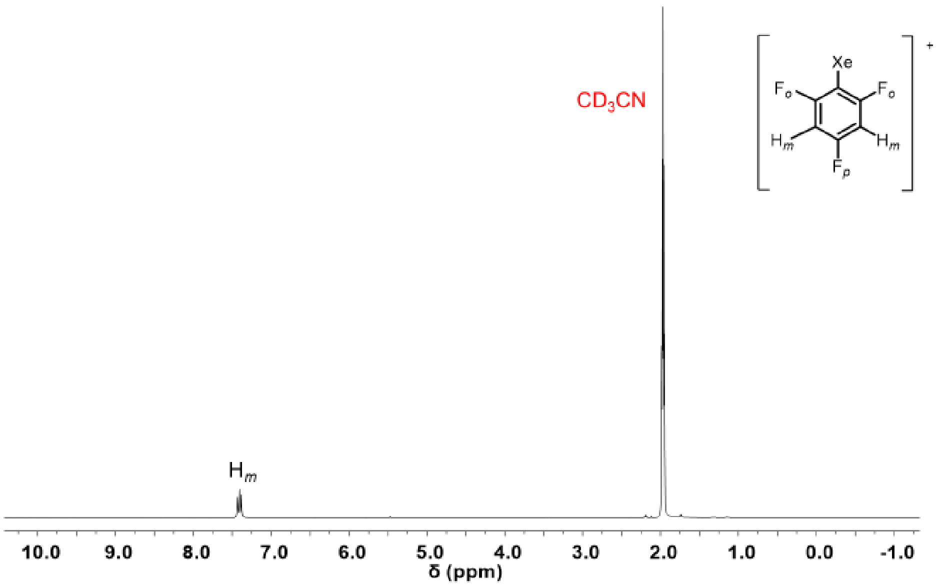

Figure S25. ^{1}H NMR spectrum (300.13 MHz, CD_3CN, 298 K) of $[(2,4,6\text{-}C_6H_2F_3)Xe][BF_4]$ (**3-Xe**).

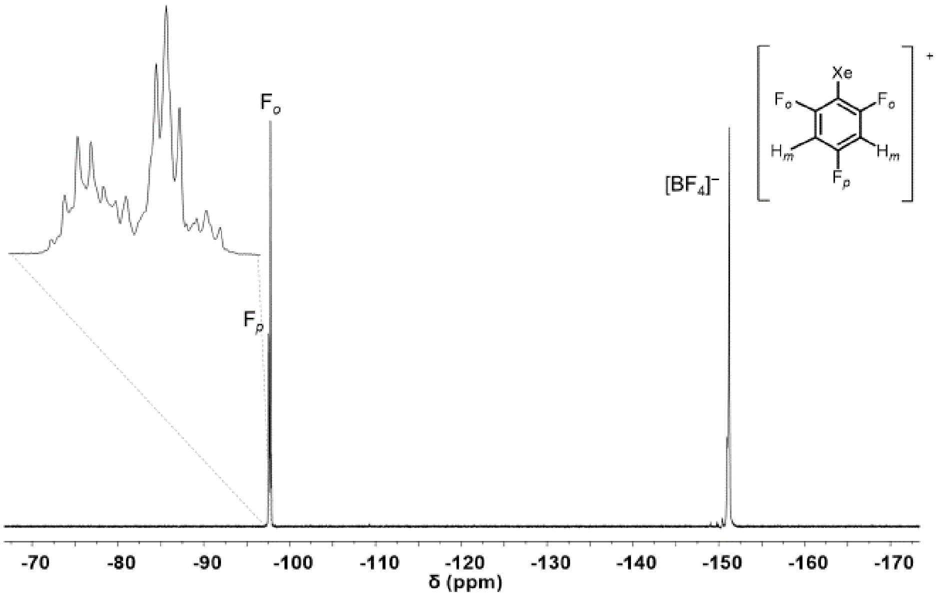

Figure S26. ^{19}F NMR spectrum (282.40 MHz, CD_3CN, 298 K) of $[(2,4,6\text{-}C_6H_2F_3)Xe][BF_4]$ (**3-Xe**).

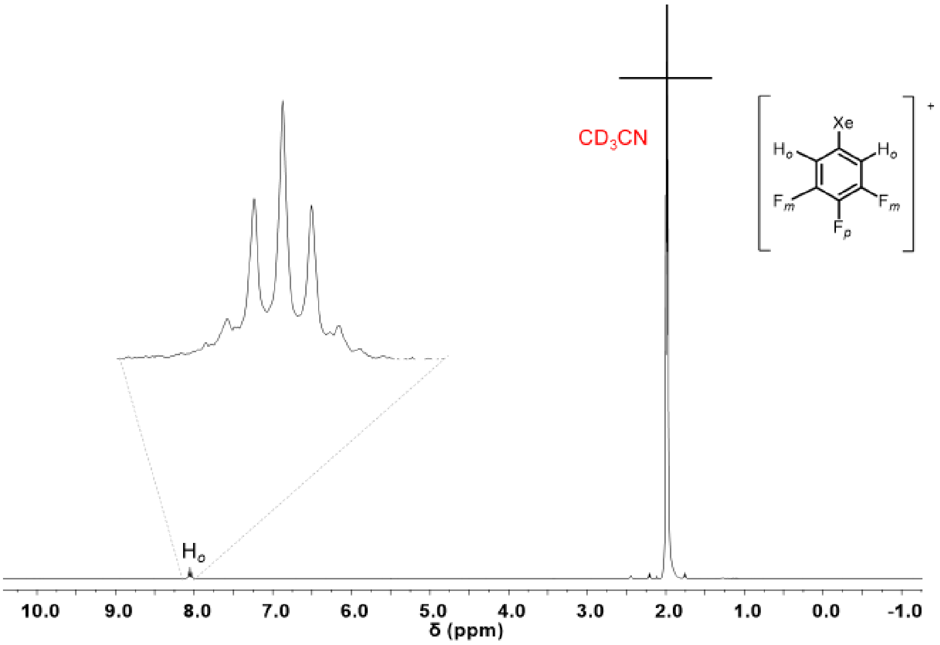

Figure S27. ^{1}H NMR spectrum (300.13 MHz, CD$_3$CN, 233 K) of [(3,4,5-$C_6H_2F_3$)Xe][BF_4] (**4-Xe**).

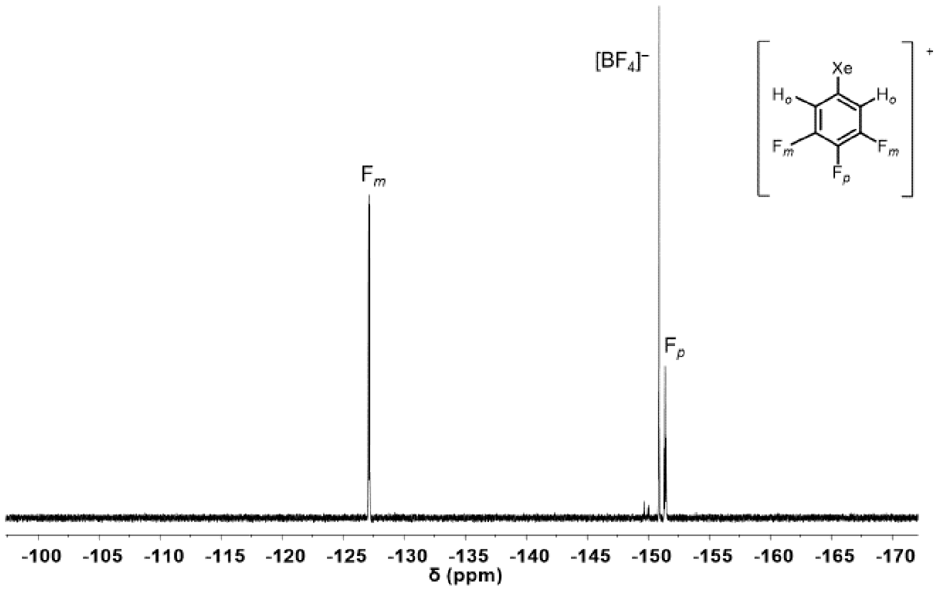

Figure S28. ^{19}F NMR spectrum (282.40 MHz, CD$_3$CN, 233 K) of [(3,4,5-$C_6H_2F_3$)Xe][BF_4] (**4-Xe**).

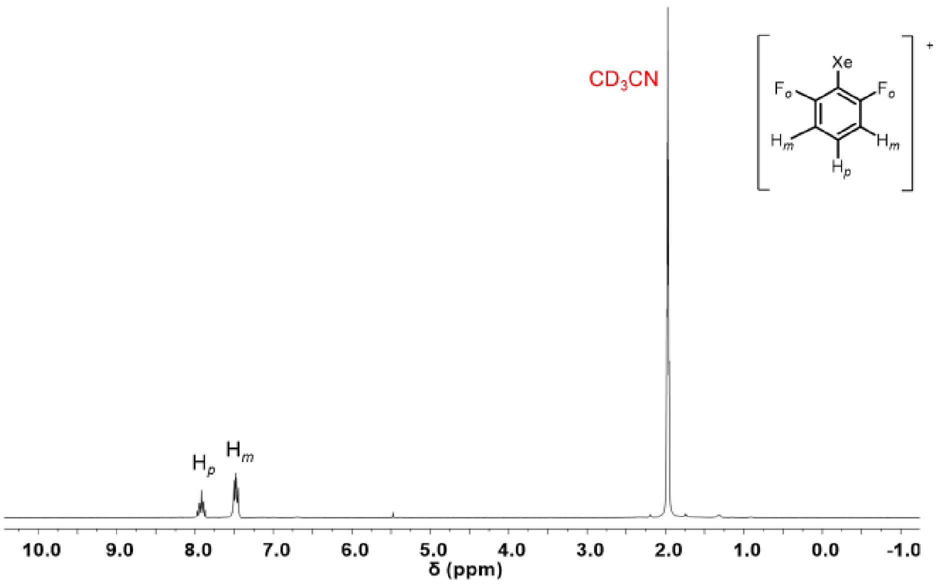

Figure S29. ^{1}H NMR spectrum (300.13 MHz, CD_3CN, 298 K) of $[(2,6\text{-}C_6H_3F_2)Xe][BF_4]$ (**5-Xe**).

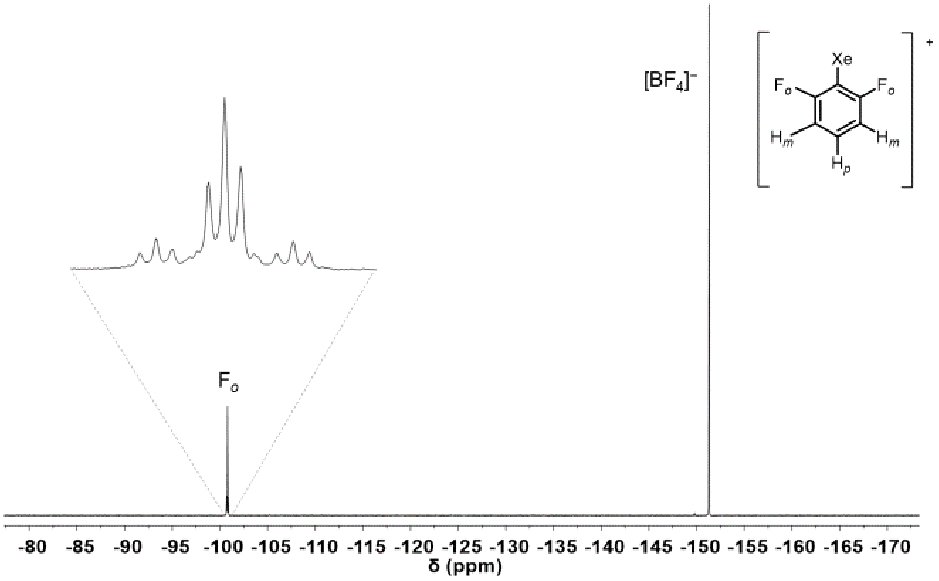

Figure S30. ^{19}F NMR spectrum (282.40 MHz, CD_3CN, 298 K) of $[(2,6\text{-}C_6H_3F_2)Xe][BF_4]$ (**5-Xe**).

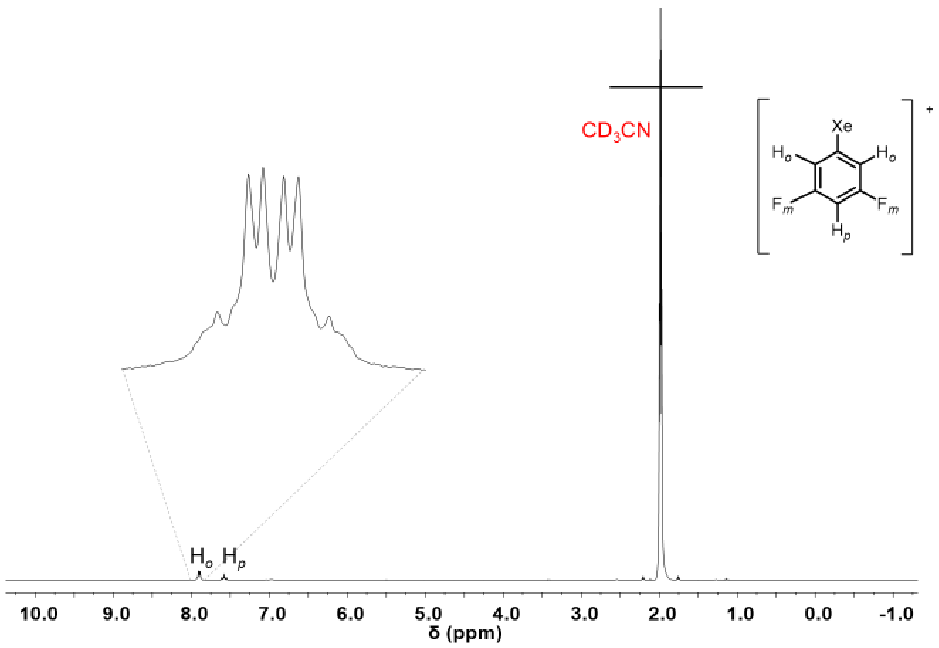

Figure S31. ^{1}H NMR spectrum (300.13 MHz, CD_3CN, 233 K) of $[(3,5\text{-}C_6H_3F_2)Xe][BF_4]$ (**6-Xe**).

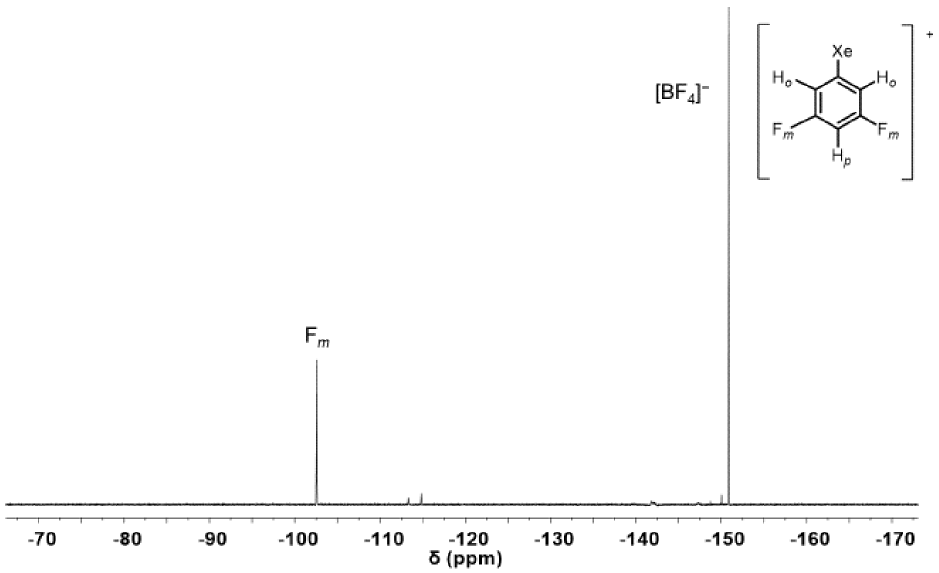

Figure S32. ^{19}F NMR spectrum (282.40 MHz, CD_3CN, 233 K) of $[(3,5\text{-}C_6H_3F_2)Xe][BF_4]$ (**6-Xe**).

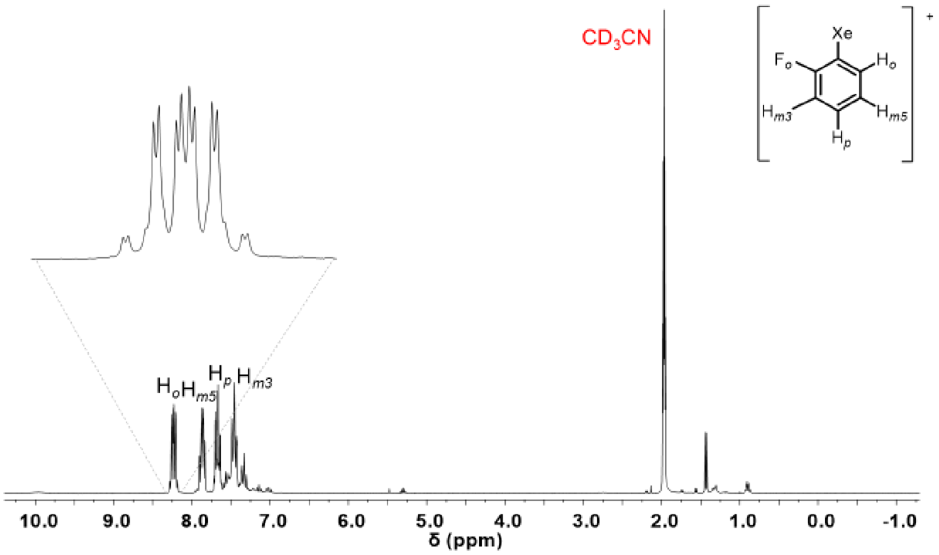

Figure S33. ^{1}H NMR spectrum (300.13 MHz, CD_3CN, 298 K) of [(2-C_6H_4F)Xe][BF_4] (**7-Xe**).

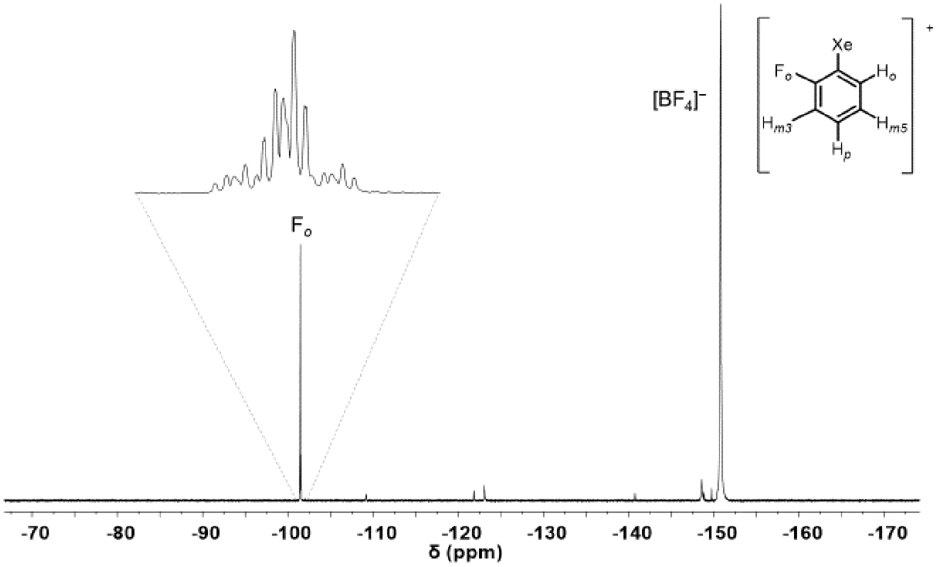

Figure S34. ^{19}F NMR spectrum (282.40 MHz, CD_3CN, 298 K) of [(2-C_6H_4F)Xe][BF_4] (**7-Xe**).

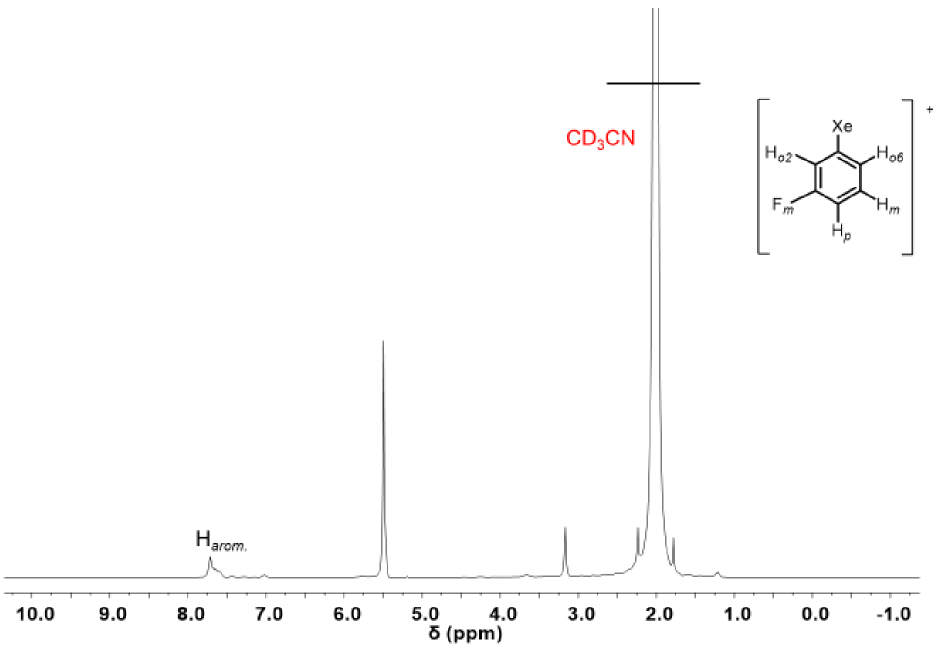

Figure S35. ^{1}H NMR spectrum (300.13 MHz, CD_3CN, 233 K) of [(3-C_6H_4F)Xe][BF_4] (**8-Xe**).

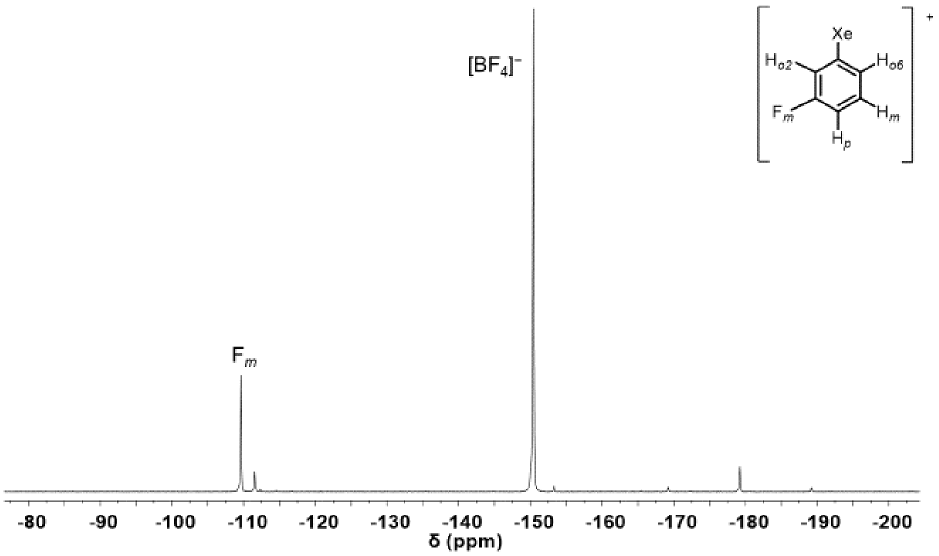

Figure S36. ^{19}F NMR spectrum (500.13 MHz, CD_3CN, 233 K) of [(3-C_6H_4F)Xe][BF_4] (**8-Xe**).

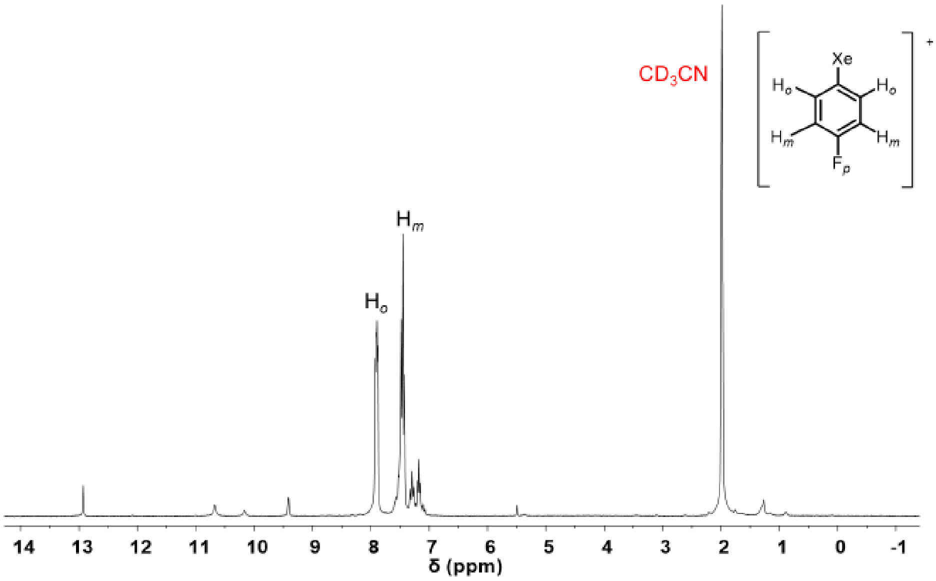

Figure S37. ^{1}H NMR spectrum (300.13 MHz, CD_3CN, 233 K) of [(4-C_6H_4F)Xe][BF_4] (**9-Xe**).

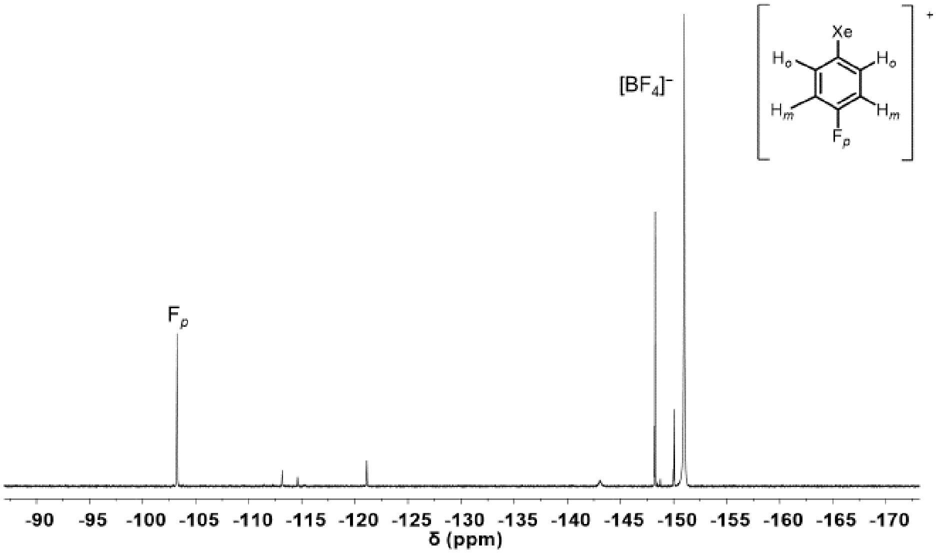

Figure S38. ^{19}F NMR spectrum (282.40 MHz, CD_3CN, 233 K) of [(4-C_6H_4F)Xe][BF_4] (**9-Xe**).

5. Quantum-chemical calculations

5.1. Computational details

Density Functional Theory (DFT) calculations were performed by using the Gaussian 16 (Revision A.03) software package.[11] Geometry optimizations were carried out by means of the B3LYP exchange-correlation functional,[12] in conjunction with the D3BJ dispersion correction scheme[13,14] and the triple-zeta def2-TZVPP Ahlrichs basis set.[15] The nature of stationary points was confirmed by a vibrational analysis calculation to be energy minima (no imaginary frequencies).

Bond dissociation energies, D_e(B−OEt_2), of the $RBF_2{\cdot}OEt_2$ adducts (R = aryl group) were calculated from the gas-phase electronic energies, similarly to Ref. 16, including thermal corrections as obtained from harmonic vibrational frequencies, and according to the equation:

$$D_\mathrm{e}(\mathrm{RBF_2 \cdot OEt_2}) = -\Delta E = E_{\mathrm{RBF_2 \cdot OEt_2}} - E_{\mathrm{Et_2O}} - E_{\mathrm{RBF_2}}$$

Fluoride Ion Affinities (FIAs) were calculated at the B3LYP-D3BJ/def2-TZVPP level of theory (frequency analyses and single-point energies) using the Me_3SiF/Me_3Si^+ system as the anchor point.[17,18]

5.2. Cartesian coordinates of the DFT-optimized structures

The coordinates of the DFT-optimized structures for the sets of compounds RBF_2, $RBF_2{\cdot}OEt_2$ and $[RBF_3]^-$ are provided below, grouped for each R moiety (R = aryl group).

R = C_6F_5

RBF_2

F	0.77057	2.36991	0.00002
C	0.15397	1.18833	0.00001
C	-1.23123	1.20283	0.00000
F	-1.90260	2.35308	-0.00000
C	-1.92433	-0.00000	-0.00001
F	-3.25033	-0.00000	-0.00001
C	-1.23123	-1.20283	0.00000
F	-1.90260	-2.35308	-0.00000
C	0.15397	-1.18833	0.00001
F	0.77057	-2.36991	0.00002
C	0.89294	0.00000	0.00002
B	2.45007	-0.00000	0.00002
F	3.13859	1.12605	-0.00002
F	3.13859	-1.12605	-0.00002

$RBF_2·OEt_2$

F	-3.01257	2.29901	-0.20181
C	-2.34658	1.15039	-0.05452
C	-2.96838	-0.04951	-0.36157
F	-4.22610	-0.06157	-0.79987
C	-2.26686	-1.23607	-0.21766
F	-2.85569	-2.39575	-0.52257
C	-0.95636	-1.20164	0.23641
F	-0.33357	-2.38951	0.33253
C	-0.29274	-0.02375	0.56954
B	1.22650	-0.01038	1.13822
C	-1.03471	1.14207	0.39723
F	-0.49268	2.34387	0.65791
F	1.64441	-1.18366	1.69327
F	1.56099	1.09639	1.85888
O	2.24174	0.12131	-0.20259
C	2.51523	-1.09335	-0.96866
C	3.85166	-1.67773	-0.57676
H	2.47522	-0.82219	-2.02216
H	1.70427	-1.78360	-0.75654
H	4.66719	-0.98848	-0.79059
H	4.02064	-2.59713	-1.13947
H	3.86008	-1.91621	0.48437
C	2.19717	1.36258	-0.95846
H	1.79553	2.09376	-0.26576
H	1.49117	1.23002	-1.78029
C	3.57077	1.76640	-1.44256
H	4.25580	1.87360	-0.60290
H	3.49727	2.72779	-1.95202
H	3.98801	1.04859	-2.14814

$[RBF_3]^-$

F	0.42866	2.43616	0.00000
C	-0.11400	1.20337	-0.00000
C	-1.50498	1.16852	-0.00000
F	-2.22920	2.30636	0.00000
C	-2.15900	-0.04973	-0.00000
F	-3.50403	-0.10275	-0.00000
C	-1.41125	-1.21555	-0.00000
F	-2.04416	-2.40610	0.00000
C	-0.02607	-1.13500	-0.00000
F	0.63023	-2.30985	0.00000
C	0.67725	0.06397	-0.00000
B	2.35506	0.03559	-0.00000
F	2.88039	1.33218	-0.00001
F	2.77756	-0.64975	-1.15478
F	2.77756	-0.64974	1.15479

R = 2,3,5,6-C_6HF_4

RBF_2

F	0.49858	-2.37141	-0.00000
C	-0.12123	-1.18961	-0.00000
C	-1.50770	-1.18874	-0.00000
F	-2.16583	-2.35380	-0.00000
C	-2.21255	0.00005	-0.00000
H	-3.29244	0.00008	-0.00000
C	-1.50764	1.18879	0.00000
F	-2.16571	2.35389	0.00000
C	-0.12116	1.18960	0.00000
F	0.49870	2.37137	0.00000
C	0.61551	-0.00002	0.00000
B	2.17479	-0.00005	-0.00000
F	2.86419	-1.12544	0.00001
F	2.86418	1.12536	-0.00001

$RBF_2{\cdot}OEt_2$

F	-3.24869	2.27153	-0.39448
C	-2.59171	1.11346	-0.22348
C	-3.19961	-0.07709	-0.57064
H	-4.20037	-0.09452	-0.97471
C	-2.48397	-1.24429	-0.38860
F	-3.03421	-2.42219	-0.72294
C	-1.19817	-1.21558	0.13147
F	-0.56680	-2.39843	0.25634
C	-0.56446	-0.03178	0.50242
B	0.92623	-0.00737	1.14205
C	-1.30534	1.12999	0.29634
F	-0.78428	2.33557	0.58952
F	1.33004	-1.17674	1.71563
F	1.22258	1.10138	1.87616
O	1.99821	0.13217	-0.15580
C	2.31541	-1.08120	-0.90659
C	3.64023	-1.65068	-0.45728
H	2.31698	-0.81288	-1.96159
H	1.50344	-1.77925	-0.72663
H	4.45699	-0.95349	-0.63841
H	3.84229	-2.56979	-1.00948
H	3.60613	-1.88629	0.60401
C	1.97030	1.37079	-0.91635
H	1.53150	2.09888	-0.24316
H	1.30030	1.22809	-1.76611
C	3.35805	1.78947	-1.34446
H	4.00642	1.90679	-0.47747
H	3.29471	2.74864	-1.85948
H	3.81258	1.07470	-2.02987

$[RBF_3]^-$

F	0.10216	2.43328	-0.00000
C	-0.41434	1.18752	-0.00000
C	-1.80162	1.10345	0.00000
F	-2.54282	2.23978	0.00000
C	-2.44556	-0.11755	0.00000
H	-3.52262	-0.18516	0.00000
C	-1.65218	-1.24987	-0.00000
F	-2.24410	-2.47019	0.00000
C	-0.26967	-1.15188	-0.00000
F	0.41830	-2.31044	-0.00000
C	0.40457	0.06559	-0.00000
B	2.08076	0.07828	0.00000
F	2.57896	1.38627	-0.00000
F	2.52106	-0.59657	-1.15530
F	2.52106	-0.59656	1.15530

R = 2,4,6-$C_6H_2F_3$

RBF_2

F	0.42414	2.35751	0.00004
C	-0.21049	1.17908	0.00003
C	-1.59156	1.21485	0.00001
H	-2.12716	2.15104	0.00000
C	-2.25330	-0.00002	-0.00001
F	-3.59216	-0.00004	-0.00003
C	-1.59153	-1.21487	-0.00001
H	-2.12708	-2.15108	-0.00003
C	-0.21047	-1.17905	0.00002
F	0.42421	-2.35745	0.00001
C	0.54785	0.00001	0.00003
B	2.09924	-0.00000	0.00002
F	2.79497	1.12445	-0.00009
F	2.79495	-1.12446	0.00002

$RBF_2 \cdot OEt_2$

H	3.24355	-2.10273	-0.20759
C	2.71574	-1.16519	-0.12915
C	3.29473	0.02848	-0.51310
F	4.54934	0.01557	-0.99800
C	2.62700	1.23415	-0.42275
H	3.08576	2.16108	-0.72940
C	1.33765	1.20362	0.08162
F	0.70045	2.39344	0.14042
C	0.66962	0.05659	0.50966
B	-0.81681	0.07645	1.13513
C	1.42323	-1.10844	0.36523
F	0.87484	-2.29128	0.71554
F	-1.23233	1.27500	1.63554
F	-1.14310	-0.99123	1.91583
O	-1.89837	-0.12642	-0.18036
C	-2.18892	1.04786	-0.99601
C	-3.51527	1.65259	-0.59879
H	-2.17666	0.72952	-2.03723
H	-1.37327	1.74718	-0.83776
H	-4.33610	0.95507	-0.75985
H	-3.69892	2.54594	-1.19784
H	-3.49663	1.93842	0.45056
C	-1.85704	-1.39723	-0.87921
H	-1.43661	-2.09390	-0.16222
H	-1.16677	-1.29947	-1.71943
C	-3.23586	-1.83402	-1.32009
H	-3.90489	-1.90812	-0.46400
H	-3.16452	-2.81667	-1.78777
H	-3.67215	-1.15119	-2.04872

$[RBF_3]^-$

F	0.08888	2.41579	-0.00000
C	-0.47530	1.18528	-0.00000
C	-1.86894	1.18498	-0.00000
H	-2.42640	2.10916	0.00000
C	-2.49703	-0.04113	0.00000
F	-3.85920	-0.08590	0.00000
C	-1.79025	-1.22605	-0.00000
H	-2.28532	-2.18505	0.00000
C	-0.40360	-1.12873	-0.00000
F	0.25789	-2.30815	-0.00000
C	0.33020	0.05390	-0.00000
B	1.99718	0.01472	0.00000
F	2.54146	1.30743	-0.00000
F	2.42745	-0.67387	-1.15484
F	2.42745	-0.67387	1.15484

R = 3,4,5-$C_6H_2F_3$

RBF_2

H	0.82949	-2.15868	0.00006
C	0.31386	-1.20919	0.00005
C	-1.06549	-1.19807	0.00001
F	-1.76033	-2.34207	-0.00002
C	-1.77042	-0.00002	-0.00001
F	-3.10011	-0.00005	-0.00005
C	-1.06554	1.19809	0.00001
F	-1.76045	2.34203	-0.00000
C	0.31379	1.20927	0.00005
H	0.82942	2.15876	0.00007
C	1.01936	0.00006	0.00007
B	2.56513	0.00002	0.00011
F	3.25718	-1.13035	-0.00004
F	3.25727	1.13034	-0.00009

$RBF_2 \cdot OEt_2$

F	-2.94847	2.34670	-0.07158
C	-2.30105	1.17786	0.06412
C	-2.95616	0.01550	-0.31861
F	-4.19683	0.06181	-0.81147
C	-2.29652	-1.19586	-0.16901
F	-2.93874	-2.31893	-0.52906
C	-1.01306	-1.25184	0.34013
H	-0.54812	-2.21986	0.46362
C	-0.34949	-0.08003	0.71425
B	1.14034	-0.13373	1.26567
C	-1.01846	1.13946	0.57524
H	-0.55887	2.06846	0.88176
F	1.58686	-1.35840	1.66772
F	1.53863	0.91352	2.04197
O	2.17769	0.11814	-0.14563
C	2.30793	-0.99717	-1.06967
C	3.59581	-1.74760	-0.82065
H	2.25681	-0.59594	-2.08079
H	1.43907	-1.63283	-0.91724
H	4.46373	-1.11124	-0.98825
H	3.65705	-2.59958	-1.49963
H	3.62437	-2.11686	0.20211
C	2.18133	1.43691	-0.74274
H	1.88777	2.10430	0.06082
H	1.41459	1.46484	-1.51931
C	3.54854	1.80614	-1.27514
H	4.29310	1.76064	-0.48163
H	3.51764	2.82556	-1.66145
H	3.86344	1.15351	-2.08902

$[RBF_3]^-$

H	0.55868	2.14595	-0.04043
C	0.03897	1.19755	-0.02114
C	-1.34148	1.18910	-0.00429
F	-2.04591	2.34977	-0.00206
C	-2.05398	-0.00003	0.00570
F	-3.40492	-0.00007	0.01773
C	-1.34139	-1.18911	-0.00429
F	-2.04572	-2.34984	-0.00206
C	0.03907	-1.19744	-0.02115
H	0.55878	-2.14584	-0.04043
C	0.76132	0.00009	-0.02631
B	2.40035	0.00002	-0.00027
F	2.85276	-0.00012	1.34261
F	2.89228	1.15711	-0.64961
F	2.89216	-1.15698	-0.64982

R = 2,6-$C_6H_3F_2$

RBF_2

F	-0.06651	2.35858	-0.00002
C	0.57270	1.17920	-0.00001
C	1.95436	1.20693	0.00001
H	2.46702	2.15694	0.00002
C	2.64029	-0.00011	0.00002
H	3.72134	-0.00015	0.00004
C	1.95425	-1.20707	0.00001
H	2.46683	-2.15714	0.00002
C	0.57259	-1.17922	-0.00001
F	-0.06676	-2.35852	-0.00002
C	-0.18432	0.00002	-0.00002
B	-1.73782	0.00005	-0.00002
F	-2.43476	1.12399	0.00002
F	-2.43478	-1.12387	0.00002

RBF_2·OEt_2

H	3.48610	-2.09857	-0.57378
C	2.98464	-1.14878	-0.46113
C	3.54617	0.03775	-0.91026
H	4.51563	0.02957	-1.38836
C	2.86258	1.23439	-0.74937
H	3.26713	2.17569	-1.09073
C	1.62530	1.20947	-0.12972
F	0.98688	2.39854	-0.01415
C	1.00414	0.06140	0.36149
B	-0.42094	0.07856	1.11666
C	1.74376	-1.10246	0.15052
F	1.22819	-2.28708	0.55423
F	-0.79827	1.27574	1.64934
F	-0.67913	-0.98840	1.92322
O	-1.61116	-0.13007	-0.10568
C	-1.97207	1.04205	-0.89531
C	-3.26065	1.64508	-0.38653
H	-2.04925	0.72194	-1.93327
H	-1.14704	1.74299	-0.80910
H	-4.09085	0.94558	-0.47515
H	-3.49710	2.53714	-0.96881
H	-3.15220	1.93256	0.65692
C	-1.62040	-1.40078	-0.80516
H	-1.13489	-2.09402	-0.12703
H	-1.00496	-1.29909	-1.70118
C	-3.02902	-1.84705	-1.12645
H	-3.62146	-1.92592	-0.21605
H	-2.99176	-2.82902	-1.59956
H	-3.53102	-1.16663	-1.81395

$[RBF_3]^-$

F	0.31853	2.40269	-0.00000
C	0.85624	1.15647	0.00000
C	2.24640	1.11202	0.00000
H	2.80488	2.03787	0.00000
C	2.87508	-0.12415	0.00000
H	3.95571	-0.18683	0.00000
C	2.10631	-1.28092	0.00000
H	2.55212	-2.26591	-0.00000
C	0.72456	-1.15621	-0.00000
F	0.02719	-2.31901	-0.00000
C	0.02070	0.04567	-0.00000
B	-1.64614	0.04958	0.00000
F	-2.16150	1.35483	-0.00000
F	-2.09532	-0.62760	1.15530
F	-2.09532	-0.62760	-1.15530

R = 3,5-$C_6H_3F_2$

RBF_2

H	-0.51607	-2.15911	0.00003
C	0.00046	-1.21050	0.00002
C	1.38158	-1.18268	0.00001
F	2.06287	-2.34267	0.00001
C	2.10156	0.00016	-0.00000
H	3.18111	0.00029	-0.00001
C	1.38126	1.18281	-0.00001
F	2.06227	2.34297	-0.00002
C	0.00014	1.21027	0.00000
H	-0.51667	2.15872	0.00000
C	-0.70273	-0.00023	0.00002
B	-2.25087	-0.00017	0.00003
F	-2.94430	-1.12989	-0.00005
F	-2.94391	1.12982	0.00002

$RBF_2{\cdot}OEt_2$

F	-3.17547	2.37501	-0.30535
C	-2.55983	1.18559	-0.13239
C	-3.22053	0.04213	-0.54696
H	-4.20132	0.08543	-0.99513
C	-2.56617	-1.16009	-0.34526
F	-3.18718	-2.29603	-0.72872
C	-1.31212	-1.24075	0.23168
H	-0.86273	-2.21123	0.38682
C	-0.65986	-0.07098	0.63289
B	0.80006	-0.13455	1.26062
C	-1.30662	1.15519	0.44946
H	-0.85416	2.08125	0.77385
F	1.22082	-1.36107	1.68394
F	1.16899	0.90965	2.05520
O	1.90432	0.11038	-0.10279
C	2.05917	-1.00269	-1.02554
C	3.32257	-1.77434	-0.72171
H	2.06073	-0.59626	-2.03590
H	1.17420	-1.62487	-0.91640
H	4.20723	-1.15121	-0.84641
H	3.40140	-2.62449	-1.40121
H	3.29845	-2.14811	0.29953
C	1.95283	1.43137	-0.69264
H	1.63034	2.09914	0.09938
H	1.22360	1.47289	-1.50388
C	3.34838	1.78420	-1.15865
H	4.05424	1.72543	-0.33131
H	3.34945	2.80545	-1.54144
H	3.69244	1.13035	-1.95968

$[RBF_3]^-$

H	-0.24906	-2.14678	-0.03957
C	0.27156	-1.19928	-0.01972
C	1.65096	-1.17433	-0.00074
F	2.34419	-2.35159	0.00331
C	2.38510	0.00000	0.01022
H	3.46445	0.00000	0.02149
C	1.65096	1.17433	-0.00074
F	2.34419	2.35159	0.00331
C	0.27156	1.19928	-0.01972
H	-0.24907	2.14677	-0.03957
C	-0.45046	-0.00000	-0.02613
B	-2.08962	0.00000	-0.00192
F	-2.54194	-0.00015	1.34158
F	-2.58412	-1.15634	-0.65155
F	-2.58411	1.15649	-0.65129

R = 2-C_6H_4F

RBF_2

F	0.05956	2.12848	0.00000
C	-0.56775	0.93866	-0.00000
C	-1.95102	0.94562	0.00001
H	-2.47743	1.88899	0.00002
C	-2.62302	-0.26914	0.00001
H	-3.70453	-0.27819	0.00002
C	-1.91411	-1.46695	-0.00000
H	-2.44116	-2.41043	-0.00000
C	-0.52936	-1.44237	-0.00001
H	0.02488	-2.37071	-0.00002
C	0.18789	-0.23417	-0.00002
B	1.73441	-0.26375	-0.00005
F	2.49144	0.81983	0.00000
F	2.37238	-1.43063	0.00003

$RBF_2 \cdot OEt_2$

H	-4.07686	1.87727	0.29847
C	-3.37198	1.07539	0.12629
C	-3.75434	-0.04384	-0.60329
H	-4.75592	-0.12133	-1.00403
C	-2.84443	-1.07153	-0.82117
H	-3.10561	-1.95554	-1.38534
C	-1.57295	-0.94505	-0.29395
F	-0.69679	-1.96095	-0.53962
C	-1.13741	0.14588	0.44434
B	0.32645	0.26868	1.07669
C	-2.08160	1.16138	0.63596
H	-1.78662	2.03352	1.20370
F	0.71809	-0.73782	1.91965
F	0.62165	1.52505	1.54436
O	1.44328	0.10336	-0.19016
C	2.25584	-1.09740	-0.32801
C	3.55164	-0.96077	0.43799
H	2.41917	-1.24526	-1.39450
H	1.65259	-1.91730	0.04600
H	4.15727	-0.13760	0.06161
H	4.12590	-1.88369	0.34070
H	3.34794	-0.79292	1.49338
C	1.26879	0.92466	-1.36683
H	0.47681	1.62041	-1.10709
H	0.90960	0.28319	-2.17345
C	2.53860	1.65770	-1.73819
H	2.88069	2.27196	-0.90666
H	2.33932	2.30826	-2.59073
H	3.33786	0.97378	-2.02254

$[RBF_3]^-$

H	-2.34598	-0.07714	0.00000
C	-1.49493	0.59196	0.00000
C	-1.69843	1.97043	0.00000
H	-2.70499	2.37214	0.00000
C	-0.60631	2.82951	0.00000
H	-0.74559	3.90375	0.00000
C	0.67834	2.29581	0.00000
H	1.55682	2.92800	0.00000
C	0.82952	0.91756	0.00000
F	2.10941	0.45507	0.00000
C	-0.22240	0.01078	0.00000
B	-0.02610	-1.62373	0.00000
F	-1.30437	-2.24140	0.00000
F	0.67834	-2.03486	1.15418
F	0.67834	-2.03486	-1.15418

R = 3-C_6H_4F

RBF_2

H	0.17396	-1.90911	-0.00000
C	0.44985	-0.86407	-0.00000
C	1.78397	-0.51057	0.00000
F	2.71950	-1.48217	0.00000
C	2.19798	0.81078	0.00000
H	3.25437	1.03873	0.00000
C	1.23341	1.81201	0.00000
H	1.54136	2.84840	0.00000
C	-0.11504	1.48322	-0.00000
H	-0.86041	2.26621	-0.00000
C	-0.52323	0.14351	-0.00000
B	-2.02433	-0.22455	-0.00000
F	-2.43248	-1.48746	0.00000
F	-2.97027	0.70622	0.00000

$RBF_2·OEt_2$

H	2.89941	-2.76132	-0.59824
C	2.55545	-1.76267	-0.36412
C	3.35261	-0.67118	-0.69128
H	4.31024	-0.78652	-1.17833
C	2.89364	0.59096	-0.36260
F	3.66492	1.66153	-0.66456
C	1.68013	0.79861	0.26571
H	1.38457	1.80684	0.52195
C	0.86808	-0.29415	0.58513
B	-0.54548	-0.07457	1.26860
C	1.33137	-1.57522	0.26597
H	0.73368	-2.43700	0.53090
F	-0.77878	1.15129	1.81619
F	-1.07333	-1.12467	1.95589
O	-1.71466	-0.00453	-0.10297
C	-1.70612	1.20934	-0.89921
C	-2.83242	2.12682	-0.48126
H	-1.78218	0.92169	-1.94707
H	-0.73511	1.67622	-0.75104
H	-3.80356	1.66186	-0.64696
H	-2.78748	3.04853	-1.06370
H	-2.74035	2.37840	0.57308
C	-1.95049	-1.23158	-0.82848
H	-1.72160	-2.02056	-0.11934
H	-1.23810	-1.28853	-1.65394
C	-3.38413	-1.33474	-1.30294
H	-4.07116	-1.26892	-0.46034
H	-3.53128	-2.29742	-1.79414
H	-3.63643	-0.55484	-2.02118

$[RBF_3]^-$

H	0.52815	2.30381	-0.03403
C	-0.19901	1.50176	-0.01582
C	-1.55516	1.80546	0.00163
H	-1.88451	2.83839	0.00547
C	-2.50625	0.78681	0.01106
H	-3.56893	0.98715	0.02237
C	-2.04338	-0.51535	-0.00040
F	-2.96811	-1.52716	0.00215
C	-0.69945	-0.83415	-0.01809
H	-0.39448	-1.87215	-0.03699
C	0.26409	0.17982	-0.02298
B	1.86349	-0.16675	-0.00202
F	2.34594	-0.12246	1.33226
F	2.57903	0.78345	-0.77312
F	2.09174	-1.46377	-0.52564

R = 4-C_6H_4F

RBF_2

H	0.44574	2.14318	0.00056
C	-0.09065	1.20404	0.00042
C	-1.47642	1.21484	0.00005
H	-2.04182	2.13543	-0.00010
C	-2.14156	-0.00002	-0.00012
F	-3.48640	-0.00006	-0.00048
C	-1.47632	-1.21482	0.00005
H	-2.04164	-2.13546	-0.00010
C	-0.09055	-1.20391	0.00042
H	0.44593	-2.14299	0.00056
C	0.62738	0.00011	0.00061
B	2.16792	0.00003	0.00104
F	2.86783	1.12903	-0.00057
F	2.86756	-1.12916	-0.00058

$RBF_2 \cdot OEt_2$

H	-3.24351	2.12938	0.29486
C	-2.72184	1.18679	0.20825
C	-3.32529	0.12627	-0.44340
F	-4.55765	0.29980	-0.97059
C	-2.71347	-1.10761	-0.56483
H	-3.22811	-1.91430	-1.06730
C	-1.44552	-1.27139	-0.01950
H	-0.96726	-2.23966	-0.09002
C	-0.78462	-0.22736	0.63645
B	0.67100	-0.42026	1.22019
C	-1.45469	0.99618	0.74488
H	-0.98401	1.81488	1.27330
F	1.12792	-1.69771	1.33848
F	1.07126	0.43095	2.20359
O	1.78541	0.14865	-0.11173
C	1.91596	-0.73091	-1.25683
C	3.18591	-1.54539	-1.15816
H	1.89560	-0.11721	-2.15677
H	1.03404	-1.36788	-1.26809
H	4.06732	-0.90515	-1.16617
H	3.24896	-2.22974	-2.00584
H	3.18665	-2.12933	-0.24026
C	1.78500	1.56220	-0.40281
H	1.47859	2.03593	0.52453
H	1.02574	1.76141	-1.16214
C	3.15481	2.04866	-0.82616
H	3.89176	1.83455	-0.05321
H	3.12134	3.12767	-0.98222
H	3.48339	1.58947	-1.75841

$[RBF_3]^-$

H	0.16004	2.12982	-0.03778
C	-0.37751	1.18971	-0.01959
C	-1.77144	1.20778	-0.00499
H	-2.33309	2.13322	-0.00384
C	-2.44105	0.00000	0.00390
F	-3.81191	0.00000	0.01662
C	-1.77145	-1.20778	-0.00507
H	-2.33309	-2.13321	-0.00397
C	-0.37752	-1.18971	-0.01966
H	0.16004	-2.12982	-0.03791
C	0.35725	-0.00000	-0.02388
B	1.99136	-0.00000	-0.00031
F	2.45881	0.00005	1.34129
F	2.49208	1.15695	-0.65118
F	2.49209	-1.15700	-0.65109

R = C_6H_5

RBF_2

H	-0.01052	2.14297	-0.00004
C	0.52825	1.20486	-0.00003
C	1.91554	1.20601	0.00001
H	2.45759	2.14193	0.00003
C	2.60876	-0.00013	0.00003
H	3.69071	-0.00030	0.00006
C	1.91528	-1.20612	0.00001
H	2.45703	-2.14220	0.00003
C	0.52797	-1.20463	-0.00003
H	-0.01097	-2.14264	-0.00004
C	-0.18759	0.00020	-0.00004
B	-1.73035	0.00006	-0.00006
F	-2.43248	1.12872	0.00003
F	-2.43211	-1.12885	0.00003

$RBF_2 \cdot OEt_2$

H	-3.49173	2.26200	-0.26048
C	-3.01690	1.28953	-0.24960
C	-3.61202	0.21831	-0.90707
H	-4.54687	0.35571	-1.43428
C	-3.00697	-1.03259	-0.87233
H	-3.47336	-1.87329	-1.36922
C	-1.80748	-1.20457	-0.19125
H	-1.35503	-2.18738	-0.15128
C	-1.18337	-0.13838	0.46527
B	0.20036	-0.33224	1.20433
C	-1.81814	1.10808	0.42891
H	-1.37390	1.94457	0.95323
F	0.60528	-1.60960	1.44660
F	0.53482	0.57062	2.16584
O	1.45530	0.12006	-0.05102
C	1.66114	-0.83596	-1.12102
C	2.88942	-1.67572	-0.85229
H	1.74420	-0.28261	-2.05586
H	0.76342	-1.44811	-1.17513
H	3.78817	-1.06091	-0.81671
H	3.00852	-2.41528	-1.64593
H	2.78623	-2.19864	0.09613
C	1.52099	1.51044	-0.43106
H	1.14454	2.05160	0.43130
H	0.83944	1.67563	-1.26819
C	2.93702	1.93699	-0.75586
H	3.59413	1.75805	0.09424
H	2.94882	3.00378	-0.98308
H	3.33620	1.40961	-1.62222

$[RBF_3]^-$

H	-0.25787	2.12972	-0.03634
C	-0.79812	1.19063	-0.01685
C	-2.19003	1.19963	0.00139
H	-2.72817	2.14177	0.00426
C	-2.89553	-0.00001	0.01251
H	-3.97960	-0.00001	0.02522
C	-2.19002	-1.19964	0.00139
H	-2.72815	-2.14178	0.00426
C	-0.79810	-1.19062	-0.01685
H	-0.25785	-2.12971	-0.03635
C	-0.06286	0.00001	-0.02304
B	1.57152	0.00000	-0.00217
F	2.04017	-0.00013	1.33976
F	2.07447	1.15642	-0.65318
F	2.07446	-1.15629	-0.65341

R = F

RBF_2

B	-0.00000	-0.00000	0.00000
F	-0.00000	-0.00000	1.31564
F	0.00000	1.13938	-0.65782
F	-0.00000	-1.13938	-0.65782

$RBF_2 \cdot OEt_2$

F	-1.74793	-0.44156	1.18359
B	-1.33935	-0.13558	-0.08579
F	-1.67634	1.11935	-0.49779
F	-1.54230	-1.12529	-0.99888
O	0.32489	-0.05746	0.02812
C	0.85820	1.13762	0.68258
C	1.33912	2.12780	-0.35058
H	1.66032	0.81089	1.34123
H	0.05433	1.54255	1.29203
H	2.14682	1.71076	-0.95098
H	1.70942	3.02276	0.15182
H	0.52168	2.41394	-1.00844
C	0.99506	-1.30888	0.35298
H	0.32700	-2.08150	-0.01257
H	1.05835	-1.38431	1.43904
C	2.34809	-1.38959	-0.31538
H	2.24704	-1.30037	-1.39603
H	2.80003	-2.35613	-0.08989
H	3.02887	-0.61529	0.03805

$[RBF_3]^-$

B	0.00000	0.00000	-0.00001
F	0.26202	-0.22412	1.36844
F	1.13541	0.57679	-0.60803
F	-0.29797	-1.22758	-0.62911
F	-1.09946	0.87492	-0.13130

6. References

(1) Frohn, H.-J.; Franke, H.; Fritzen, P.; Bardin, V. V. (Fluoroorgano)Fluoroboranes and -Fluoroborates I: Synthesis and Spectroscopic Characterization of Potassium Fluoroaryltrifluoroborates and Fluoroaryldifluoroboranes. *J. Organomet. Chem.* **2000**, *598* (1), 127–135. https://doi.org/10.1016/S0022-328X(99)00690-7.

(2) Frohn, H.-J.; Adonin, N. Yu.; Bardin, V. V.; Starichenko, V. F. A New Application of (Polyfluoroorgano)Trifluoroborate Salts: The Palladium-Catalysed Cross-Coupling Reaction with Substituted Benzenediazonium Tetrafluoroborates. *J. Fluorine Chem.* **2002**, *117* (2), 115–120. https://doi.org/10.1016/S0022-1139(02)00157-4.

(3) Ullrich, M.; Lough, A. J.; Stephan, D. W. Dihydrogen Activation by $B(p\text{-}C_6F_4H)_3$ and Phosphines. *Organometallics* **2010**, *29* (16), 3647–3654. https://doi.org/10.1021/om100563m.

(4) Šmalc, A.; Lutar, K. Xenon Difluoride (Modification). In *Inorganic Syntheses*; Grimes, R. N., Ed.; John Wiley: New York, 1992; Vol. 29, pp. 1–4.

(5) Harris, R. K.; Becker, E. D.; Cabral de Menezes, S. M.; Granger, P.; Hoffman, R. E.; Zilm, K. W. Further Conventions for NMR Shielding and Chemical Shifts (IUPAC Recommendations 2008). *Pure Appl. Chem.* **2008**, *80* (1), 59–84. https://doi.org/10.1351/pac200880010059.

(6) Butler, H.; Naumann, D.; Tyrra, W. Fluoroaryl xenon, iodine, and tellurium tetrafluoroborates. Syntheses, properties and spectroscopic Data. *Eur. J. Solid State Inorg. Chem.* **1992**, *29*, 739–758.

(7) Frohn, H.-J.; Franke, H.; Bardin, V. V. A Simple and Convenient Route to Arylxenon(II) Tetrafluoroborates. *Z. Naturforsch. B* **1999**, *54* (12), 1495–1498. https://doi.org/10.1515/znb-1999-1203.

(8) Naumann, D.; Butler, H.; Gnann, R.; Tyrra, W. Arylxenon Tetrafluoroborates: Compounds of Unexpected Stability. *Inorg. Chem.* **1993**, *32* (6), 861–863. https://doi.org/10.1021/ic00058a018.

(9) Frohn, H. J.; Rossbach, Chr. Kationen mit Xenon—Kohlenstoff-Bindung. 4. Salze mit monosubstituiertem Phenylxenon-Kation: Darstellung, Stabilität und Reaktivität. *Z. Anorg. Allg. Chem.* **1993**, *619* (10), 1672–1678. https://doi.org/10.1002/zaac.19936191006.

(10) Koppe, K.; Bilir, V.; Frohn, H.-J.; Mercier, H. P. A.; Schrobilgen, G. J. Syntheses, Solution Multi-NMR Characterization, and Reactivities of $[C_6F_5Xe]^+$ Salts of Weakly Coordinating Borate Anions, $[BY_4]^-$ (Y = CF_3, C_6F_5, CN, or $OTeF_5$). *Inorg. Chem.* **2007**, *46* (22), 9425–9437. https://doi.org/10.1021/ic7010138.

(11) Frisch, M. J.; Trucks, G. W.; Schlegel, H. B.; Scuseria, G. E.; Robb, M. A.; Cheeseman, J. R.; Scalmani, G.; Barone, V.; Petersson, G. A.; Nakatsuji, H.; Li, X.; Caricato, M.; Marenich, A. V.; Bloino, J.; Janesko, B. G.; Gomperts, R.; Mennucci, B.; Hratchian, H. P.; Ortiz, J. V.; Izmaylov, A. F.; Sonnenberg, J. L.; Williams-Young, D.; Ding, F.; Lipparini, F.; Egidi, F.; Goings, J.; Peng, B.; Petrone, A.; Henderson, T.; Ranasinghe, D.; Zakrzewski, V. G.; Gao, J.; Rega, N.; Zheng, G.; Liang, W.; Hada, M.; Ehara, M.; Toyota, K.; Fukuda, R.; Hasegawa, J.; Ishida, M.; Nakajima, T.; Honda, Y.; Kitao, O.; Nakai, H.; Vreven, T.; Throssell, K.; Montgomery, J. A., Jr.; Peralta, J. E.; Ogliaro, F.; Bearpark, M. J.; Heyd, J. J.; Brothers, E. N.; Kudin, K. N.; Staroverov, V. N.; Keith, T. A.; Kobayashi, R.; Normand, J.; Raghavachari, K.; Rendell, A. P.; Burant, J. C.; Iyengar, S. S.; Tomasi, J.; Cossi, M.; Millam, J. M.; Klene, M.;

Adamo, C.; Cammi, R.; Ochterski, J. W.; Martin, R. L.; Morokuma, K.; Farkas, O.; Foresman, J. B.; Fox, D. J. *Gaussian 16, revision A.03*; Gaussian, Inc.: Wallingford, CT, 2016.

(12) Becke, A. D. A New Mixing of Hartree–Fock and Local Density-Functional Theories. *J. Chem. Phys.* **1993**, *98* (2), 1372–1377. https://doi.org/10.1063/1.464304.

(13) Grimme, S.; Antony, J.; Ehrlich, S.; Krieg, H. A Consistent and Accurate *ab initio* Parametrization of Density Functional Dispersion Correction (DFT-D) for the 94 Elements H-Pu. *J. Chem. Phys.* **2010**, *132* (15), 154104. https://doi.org/10.1063/1.3382344.

(14) Johnson, E. R.; Becke, A. D. A Post-Hartree–Fock Model of Intermolecular Interactions. *J. Chem. Phys.* **2005**, *123* (2), 024101. https://doi.org/10.1063/1.1949201.

(15) Weigend, F.; Ahlrichs, R. Balanced Basis Sets of Split Valence, Triple Zeta Valence and Quadruple Zeta Valence Quality for H to Rn: Design and Assessment of Accuracy. *Phys. Chem. Chem. Phys.* **2005**, *7* (18), 3297–3305. https://doi.org/10.1039/B508541A.

(16) Trujillo-González, D. E.; González-García, G.; Hamlin, T. A.; Bickelhaupt, F. M.; Braunschweig, H.; Jiménez-Halla, J. O. C.; Solà, M. The Search for Enhanced σ-Donor Ligands to Stabilize Boron-Boron Multiple Bonds. *Eur. J. Inorg. Chem.* **2023**, *26* (9), e202200767. https://doi.org/10.1002/ejic.202200767.

(17) Erdmann, P.; Leitner, J.; Schwarz, J.; Greb, L. An Extensive Set of Accurate Fluoride Ion Affinities for *p*-Block Element Lewis Acids and Basic Design Principles for Strong Fluoride Ion Acceptors. *ChemPhysChem* **2020**, *21* (10), 987–994. https://doi.org/10.1002/cphc.202000244.

(18) Böhrer, H.; Trapp, N.; Himmel, D.; Schleep, M.; Krossing, I. From Unsuccessful H_2-Activation with FLPs Containing $B(Ohfip)_3$ to a Systematic Evaluation of the Lewis Acidity of 33 Lewis Acids Based on Fluoride, Chloride, Hydride and Methyl Ion Affinities. *Dalton Trans.* **2015**, *44* (16), 7489–7499. https://doi.org/10.1039/C4DT02822H.
